# The accuracy of diagnostic ultrasound imaging for musculoskeletal soft tissue pathology of the extremities: a comprehensive review of the literature

**DOI:** 10.1186/s12998-015-0076-5

**Published:** 2015-11-05

**Authors:** Rogan E A Henderson, Bruce F. Walker, Kenneth J. Young

**Affiliations:** Private Practice of Chiropractic, Spearwood, WA Australia; Associate Professor, Discipline of Chiropractic, School of Health Professions, Murdoch University, Murdoch, WA Australia; Senior Lecturer, Discipline of Chiropractic, School of Health Professions, Murdoch University, Murdoch, WA Australia; 253 Winterfold Road, Coolbellup, 6163 WA Australia

## Abstract

Musculoskeletal diagnostic ultrasound imaging (MSK-DUSI) has been growing outside the traditional radiology speciality. Increased use of this technology has been reported in several healthcare settings, however an apparent gap in the knowledge of the accuracy of this diagnostic technology indicated a review was warranted. We undertook a structured review of the literature to assess the accuracy of MSK-DUSI for the diagnosis of musculoskeletal soft tissue pathology of the extremities. An electronic search of the National Library of Medicine’s PubMed database (1972 to mid-2014) was conducted. All relevant systematic reviews of diagnostic studies, all diagnostic studies published after the date of the latest systematic reviews and relevant diagnostic studies outside the scope the systematic reviews that directly compared the accuracy of MSK-DUSI (the index test) to an appropriate reference standard for the target condition were included. A fundamental appraisal of the methodological quality of studies was completed. The individual sensitivity, specificity and likelihood ratio data were extracted and entered into diagnostic accuracy tables. A total of 207 individual studies were included. The results show that MSK-DUSI has acceptable diagnostic accuracy for a wide spectrum of musculoskeletal conditions of the extremities. However, there is a lack of high quality prospective experimental studies in this area and as such clinicians should interpret the results with some caution due to the potential for overestimation of diagnostic accuracy.

**Electronic supplementary material:**

The online version of this article (doi:10.1186/s12998-015-0076-5) contains supplementary material, which is available to authorized users.

## Background

Musculoskeletal ultrasound (MSK-US) is a non-ionizing imaging modality, which is relatively inexpensive, portable, safe and rapid [1–4]. MSK-US should be considered in two distinct sub-categories. 1) Musculoskeletal diagnostic ultrasound imaging (MSK-DUSI) which primarily focuses on the morphological characteristics and structural integrity of the neuromusculoskeletal system [5–7]. 2) Rehabilitative ultrasound imaging (RUSI) which evaluates muscle and related soft tissue morphology and function during exercise and physical tasks [8, 9].

Historically, diagnostic ultrasound imaging (DUSI) has been utilised in medicine since the early 1950’s [5, 7]. In the following decades, DUSI became well-established in clinical obstetrics, gynaecology and cardiology [5]. In 1972, the first clinically significant application of DUSI was used in musculoskeletal medicine; where it was used to differentiate Baker’s cysts from thrombophlebitis [10]. This paper led to the logical extension of DUSI in musculoskeletal medicine seen today. The primary use of MSK-US continues to be used for traditional diagnostic imaging purposes, allowing real-time, dynamic evaluation of neuromusculoskeletal structures, including but not limited to joints, tendons, ligaments, muscles and nerves [5–7].

In the 1980’s, a new branch of MSK-US was developing. Young and colleagues used MSK-US to accurately measure muscle atrophy post-injury, aging on muscle size and the relationship between muscle size and strength in different populations [11–13]. This work established RUSI [7]. In the past two decades, a series of studies highlighted the use of RUSI to detect abnormal lumbar multifidus and transverse abdominus muscle morphology and activation patterns in patients with acute and chronic lower back pain [7]. In addition, researchers utilising RUSI found that recovery of these muscles was not automatic when pain subsided, but required specific training to re-activate them [7]. This has cemented RUSI, particularly in physiotherapy practice, where RUSI has been used to monitor the recovery of these muscles [7]. Concurrently, adult spinal MSK-DUSI created a high degree of interest among groups of chiropractors for the evaluation of spinal canal diameters, facet, intervertebral disc and nerve root pathology [14–21]. However, this was soon abandoned due to technological factors such as inadequate depth of penetration, lack of penetration of spinal structures and poor image resolution resulting in limited accuracy and clinical utility [14, 22–30].

In the past decade, interest in MSK-DUSI among non-radiologists has made a resurgence [31] and has attracted the attention of many chiropractic practitioners. The use of MSK-DUSI in clinical practice has nearly quadrupled in the United States since the 2000’s and is also an expanding area in Europe, driven primarily by increased utilisation by non-radiologists [3, 31]. This can be attributed to recent advances in conventional two-dimensional (2D) ultrasound technology [32–36]. Recent refinements in 2D ultrasound technology, such as broadband transducers are now available at frequencies greater than 15 MHz that allow visualization of superficial and deep structures with resolutions approaching that of standard T1 and T2 magnetic resonance imaging (MRI) sequences [33]. Ultrasound machines are now available as compact, portable systems (typically the size of a notebook computer), which are on average available at less cost than the conventional cart-based systems. [32, 33] Although the cart-based systems allow for extra features including: greater image and patient information archive capacity and more ultrasound mode options (i.e. 2D, Doppler, harmonic mode etc.), the mid-range compact, portable systems are cheaper and have similar image resolution [32, 34]. Reduced machine cost, portability, improved image resolution and increased diagnostic accuracy have influenced MSK-DUSI growth among non-radiologists and interest among chiropractors.

A review of the literature revealed a current paucity of studies examining the utility of MSK-DUSI within chiropractic environments. The majority consist of experimental studies assessing the normal or abnormal sonographic appearance of anatomy [37–42]; several case reports demonstrating the importance of subsequent MSK-DUSI when plain films are unremarkable [43–46]; and a few commentaries [14, 16]. One pilot study by Hung et al. [47] showed that it may be feasible to teach senior chiropractic students an area of normal sonographic anatomy. This is consistent with other professions demonstrating an ability to train novice interpreters to a standard of that of an experienced interpreter [48–51]. Extrapolations of these studies suggest it may be feasible to teach the sonographic appearance of other body regions to novice interpreters. Although the literature on the topic is limited, current observational trends of increasing accessibility to MSK-DUSI training for chiropractors suggest that a growing body of literature may emerge.

MSK-DUSI has been reported as a valid technique for imaging a wide variety of neuromusculoskeletal conditions [52]. However, it is important to emphasise with the current state of the technology the utility of MSK-DUSI is typically limited to the diagnosis of superficial pathology of the extremities. MSK-DUSI has little use in the spine other than landmark identification for injection purposes by medical professionals and research. Nonetheless, a high percentage of chiropractic patients undergoing diagnosis and treatment have musculoskeletal complaints of the extremities [53, 54]. Improving patient care is pivotal in all healthcare professions and by developing the utility of MSK-DUSI in the chiropractic profession may allow for earlier, accurate diagnosis and therefore, better patient management and outcomes. MSK-DUSI involves no ionising radiation, meaning it is safe for patients. Accessibility is increasing as costs of the systems diminish. Portability allows efficient and accurate ‘in office’ scanning as a potential extension to physical examination for certain anatomic areas. MSK-DUSI has become an accurate, prompt, relatively inexpensive and readily available method of imaging the neuromusculoskeletal system. This presents new opportunities within the chiropractic profession to improve patient care and research.

The growing appeal of MSK-DUSI among the chiropractic profession can be observed from the recent accessibility to tailored courses and through the growing number of published research papers [14–16, 37–46]. The appropriate use of imaging is essential in all healthcare professions for accurate patient diagnosis and management as well as optimising the use of healthcare resources. However, the instrument of measurement needs to be reliable and valid. Therefore, this review investigated the evidence currently available on the accuracy of diagnostic ultrasound for the diagnosis of musculoskeletal soft tissue pathology of the extremities.

## Review

### Identification and selection of studies

The anatomical areas selected included: shoulder, elbow, hand/wrist, hip, knee and ankle/foot. These areas corresponded to the MSK-DUSI guidelines identified by the European Society of Musculoskeletal Radiology (ESMR) and the American College of Radiology (ACR) [3, 55]. All clinically indicated musculoskeletal soft-tissue conditions identified by the ESMR and ACR MSK-DUSI guidelines were included this review [3, 55]. Therefore, for the purpose of this article ‘soft tissue pathology’ was defined to mean musculoskeletal conditions of muscle, tendon, ligament, and certain joint and peripheral nerve structures.

The conclusions of the report are based on the results of relevant systematic reviews of diagnostic studies, all diagnostic studies published after the date of the latest systematic reviews and relevant diagnostic studies outside the scope the systematic reviews. Articles included in the systematic reviews were not treated individually in this review but were included as a whole review. While critical appraisal of the included reviews and diagnostic studies would be ideal, it is beyond the scope of the present report.

A review of the literature was performed using the National Library of Medicine’s PubMed data base (1972 to mid-2014). The term ‘index test’ was defined to mean the test whose performance was being evaluated. The reference test or ‘gold standard’ was the standard against which the index test was compared. All systematic reviews and diagnostic studies that assessed the accuracy of MSK-DUSI (the index test) to an appropriate reference test for musculoskeletal soft tissue pathology of the extremities were included. The reference test is dependent on the target condition and includes: MRI, surgical findings (arthroscopy or open surgery), arthrography and electromyography (EMG), or nerve conduction studies (NCS). The search strategy used three important ‘search term sets’ including: index test set, target condition set, and diagnostic accuracy set. Additional file 1 shows the full electronic search strategy. The search was restricted to articles published in English or languages for which a full translation to English was also published. The titles and abstracts retrieved were screened by one reviewer (RH) to identify potentially relevant studies for inclusion and duplicates removed. Full-text manuscripts were obtained and evaluated for final inclusion against a predetermined criteria (Table 1). The reference lists of each potentially relevant paper were reviewed to identify any omitted studies missed by the search strategy.

**Table 1:** Inclusion

• Published as full-text article, published in English and languages for which a full translation could be obtained.
• Human studies only (no cadaveric studies).
• Index test: MSK-DUSI.
• Target condition(s): musculoskeletal soft tissue pathology.
• Reference tests: MRI, surgical findings (arthroscopy or open surgery), arthrography and electromyography or nerve conduction studies.
• Interpreted by radiolo
• Individual diagnostic studies not included in prior systematic review/meta-analysis.
• Sufficient quantitative data provided (minimum: two-by-two tables)

### Data extraction and analysis

A fundamental appraisal of the methodological quality of studies was completed by the reviewer (RH), as outlined by the Users’ Guide to the Medical Literature: A Manual for Evidence-Based Clinical Practice [56]. The following items were assessed:

1. Was the patient sample appropriate? (i.e. representative of clinical practice; uncertain diagnosis; wide spectrum – age, gender, severity; patients with disease/with similar presenting disease/ without disease)
2. Was there an independent, blinded comparison to an appropriate reference standard?
3. Did all patients, regardless of index test results, undergo the reference standard?

The following data were extracted:

1. Publication details.
2. Sample size.
3. Baseline characteristics: age, duration of symptoms between injury and MSK-DUSI and diagnosis.
4. Target condition as reported.
5. Index test: ultrasound transducer frequency, ultrasound operator and reviewer.
6. Reference standard.
7. Quantitative Data: Sensitivity (SnS), Specificity (SpC) and likelihood ratios (LR).

Two-by-two tables containing the number of true positives, true negatives, false positives and false negatives were the minimum quantitative data necessary for inclusion of individual diagnostic studies and systematic reviews. Articles that did not provide the required minimum quantitative data were excluded from this review. SnS, SpC and LRs were calculated from the two-by-two tables using a web-based diagnostic test calculator when they were not provided outright. [57] When SnS and SpC were provided outright without LRs, the LRs were calculated manually using an excel spreadsheet. Pooled SnS and SpC data provided from systematic reviews were extracted if provided. The authors did not pool the data. When pooled data was not provided, the data range was extracted.

The extracted data were entered into two types of tables.

1. Study characteristics tables containing: target condition; publication details; sample size; age; duration of symptoms between injury and MSK-DUSI; ultrasound transducer frequency; ultrasound operator and reviewer.
2. MSK-DUSI accuracy tables containing: target condition; publication details; reference standard; quantitative data.

### Accuracy summary tables

These tables were developed with the purpose of providing the best available evidence-based recommendations for when diagnostic ultrasound is clinically indicated for musculoskeletal soft tissue pathology. The tables are based on the results relating to each anatomical area. The Accuracy Summary is based on a five scale determination as follows. Unknown: No diagnostic accuracy studies found. Grade 0: Not indicated. Grade 1: Conflicting evidence (test results should be interpreted with caution). Grade 2: Equivalent to other imaging techniques (other techniques might provide significant information). Grade 3: First choice technique (other techniques rarely provide more information). A clinical condition received a grade 0 recommendation if the data or the majority of studies reported SnS and SpC values less than 0.60. A Grade 1 recommendation was given if there was a relatively even number of studies reporting conflicting SnS and SpC data (e.g. the positive supportive evidence does not significantly out-weigh the negative supportive evidence, and vice versa). A Grade 2 recommendation was given if the data or the majority of studies reported SnS and SpC values greater than 0.60 and less than 0.85. A Grade 3 recommendation was given if the data or the majority of studies reported SnS and SpC values greater than 0.85. The grading system was adapted from the four scale determination used in *Clinical indications for musculoskeletal ultrasound: A Delphi-based consensus paper of the European society of musculoskeletal radiology* [3].

### Data interpretation

In text, the collective diagnostic findings are classified as low, moderate and high diagnostic accuracy. There is currently no reported classification for what is considered poor, low, moderate or high diagnostic accuracy [58–60]. As such the following ranges were used to classify the collective diagnostic findings. Low (SnS and SpC: less than 0.60), moderate (SnS and SpC: 0.60 to 0.85) and high (SnS and SpC: greater than 0.85) diagnostic accuracy. These ranges reflect those of the accuracy summary tables to maintain consistency throughout the article.

It is worth remembering that tests with high SnS and small negative likelihood ratios (LR–) are most useful for ruling out disease. That is, a negative result indicates that disease is not likely to be present. Tests with high SpC and high positive likelihood ratios (LR+) are most useful for ruling in disease. That is, a positive test indicates that disease is likely to be present [61]. LRs summarise how many times more (or less) likely patients with the disease are to have a particular test result than patients without the disease. A LR+ above 10 and a LR– below 0.1 are considered to provide strong evidence to rule a diagnosis in or out, respectively [62].

## Results

**Fig. 1:**
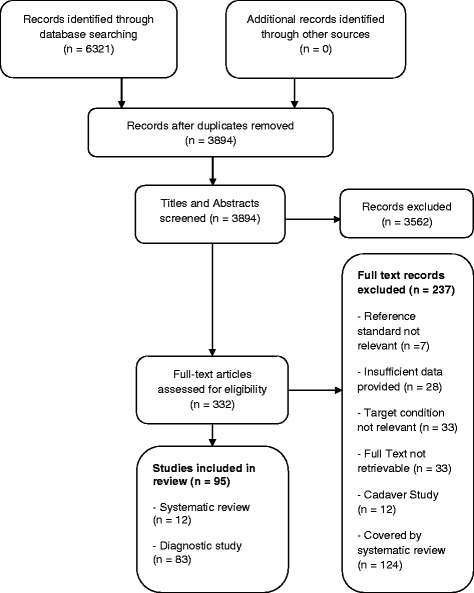
Flow chart: search strategy results

In total, the search strategy identified 6321 citations. After removing duplicates, there were 3894 potentially eligible titles and abstracts. Once the titles and abstracts of these citations were screened, 332 potentially eligible articles remained. These full-text articles were reviewed for eligibility, 95 studies (12 systematic reviews and 83 diagnostic studies) were included in the final review. The individual studies in the systematic reviews totalled 124 and when added to the other 83 diagnostic studies amounted to 207 individual studies. The full results of the search strategy are presented in a flow chart (Fig. 1).

### Shoulder

A total of 13 clinical conditions were identified (Table 2). Seventy-two diagnostic studies and five systematic reviews relevant to the accuracy of MSK-DUSI for diagnosing soft-tissue pathology of the shoulder were found. Four of the systematic reviews investigated rotator cuff tears [63–66] and one was a systematic review investigating subacromial disorders [67]. The systematic reviews contained 63 of the same articles found in this review. These articles were not treated individually in this review as they were included by way of each systematic review (refer to systematic reviews for these references). This left four relevant diagnostic studies published after the date of the latest systematic review [49, 68–70] and five relevant diagnostic studies outside the scope the systematic reviews [71–75]. Therefore, a total of 14 relevant articles were used in this review (nine diagnostic studies and five systematic reviews) [49, 63–75], amounting to 72 individual studies in all. The study characteristics are presented in Table 3.

**Table 2:** Identified clinical conditions of the shoulder

Identified clinical conditions of the shoulder	Relevant studies found (Yes/No)
Full thickness cuff tears	Y
Partial thickness cuff tears	Y
Bursitis	Y
Calcific tendinitis (Supraspinatus and long head the biceps)	Y
Rotator cuff tendinopathy (includes tendinitis and tendinosis)	Y
Rotator cuff muscle atrophy	Y
Subacromial impingement	Y
Long head of biceps: tears	Y
Long head of biceps: dislocation	Y
Long head of biceps: tendinopathy \(includes tendinitis and tendinosis)	Y
Adhesive capsulitis	N
Pectoralis tears	N
Deltoid tears	N

**Table 3:** Shoulder: Study Characteristics

Study	Target Condition	Number of studies (Systematic Review)	Subjects	Mean Age (years)	Mean time from injury to ultrasound	Ultrasound transducer frequency (MHz)	Ultrasound reviewers
Systematic Review							
Lenza et al., 2013 [65]	RC FTT/PTT	10	654	N/S	<1 year	5.0 to 15	RAD
Smith et al., 2011 [66]	RC FTT/PTT	62	6007	52.2	1 year	5.0 to 13	RAD and Non-RAD
Ottenheijm et al., 2010 [67]	RC FTT/PTT; Bursitis; CT; RCT	23	1377	52	<3 months	>7.5	RAD and Non-RAD
Kelly et al., 2009 [64]	RC FTT/PTT	67	N/S	N/S	N/S	N/S	RAD and Non-RAD
de Jesus et al., 2009 [63]	RC FTT/PTT	65	N/S	N/S	N/S	N/S	RAD
Diagnostic Study							
Alavekios et al., 2013 [68]	RC FTT	-	200	N/S	N/S	12 to 15	Non-RAD
Le Corroller et al., 2008 [69]	RC FTT/PTT; Bursitis; RCT; LHB	-	65	52.4	N/S	5.0 to 12	RAD
Murphey et al., 2013 [49]	RC FTT	-	156	N/S	1 day	4.7 to 13	RAD and Non-RAD
Ok et al., 2013 [70]	RC FTT/PTT	-	51	N/S	N/S	12	Non-RAD
Khoury et al., 2008 [72]	RC Atrophy	-	39	61	N/S	5.0 to 12	RAD
Strobel et al., 2005 [75]	RC Atrophy	-	65	53.1	N/S	7.5 to 9	RAD
Armstrong et al., 2006 [71]	LHB	-	71	59	N/S	7.5 to 9	RAD
Read et al., 1998 [73]	LHB; Impingement	-	42	44	8.8 weeks	7.5	RAD
Skendzel et al., 2011 [74]	LHB	-	66	55	<6.5 months	10 to 17	RAD
-	AC	-	-	-	-	-	-
-	Pec/Delt Tears	-	-	-	-	-	-

*N/S* not stated; *RAD* Radiologist; *RC* rotator cuff*; FTT* full tendon tear*; PTT* partial tendon tear*; CT* calcific tendonitis*; RCT* rotator cuff tendinopathy (includes tendinosis and tendinitis)*; LHB* long head of the biceps tendon; *AC* adhesive capsulitis; *Pec/Delt* pectoralis/deltoid

Table 3 reports, the five systematic reviews included a total of 227 diagnostic studies [63–67]. The number of patients was not stated in two studies [63, 64]. The other 12 studies reviewed a total of 8739 patients [49, 65–75]. The mean age was not stated in six studies [49, 63–65, 68, 70]. In the eight studies where it was stated, the mean age of the cohorts was 53.6 (SD 5.1) [66, 67, 69, 71–75]. Mean time from injury to imaging was not stated in eight studies [63, 64, 68–72, 75]. In the six studies where this was stated [49, 65–67, 73, 74], this ranged from 1 day [49] to less than 200 days [74]. All studies documented the job titles of the people who performed and reviewed the ultrasound images. In eight studies, a radiologist performed and interpreted the images [63, 65, 69, 71–75]; in four studies a radiologist and non-radiologist were involved [49, 64, 66, 67]; in two studies only a non-radiologist was involved [68, 70]. Non-radiologists were either a sonographer, physician or orthopaedic surgeon [49, 64, 66–68, 70].

The individual SnS, SpC and LRs for the ultrasound diagnosis of musculoskeletal soft-tissue pathology of the shoulder are presented in Table 4. Overall, both systematic reviews and diagnostic studies consistently demonstrated high diagnostic accuracy for full-thickness rotator cuff tears [49, 63–70]. Therefore, a positive test provides convincing evidence that a full-thickness tear is present, because it increases the odds of a full tear being present 6 to 30-fold (LR + = 6.0 to 30.0), well above the arbitrary threshold of 10 [62]. In addition, a negative test rules out a full-thickness tear, because it decreases the odds 0.04 to 0.23-fold (LR- = 0.04 to 0.23), below the 0.1 value commonly used for exclusion [62]. For partial thickness-tears, both systematic reviews and diagnostic studies results show that it is easier to rule in or diagnose patients with partial thickness tears (SpC: 0.75 to 0.98; LR + = 1.84 to 35.5) than to rule it out (SnS: 0.46 to 0.84; LR- = 0.18 to 0.72) [63–67, 69, 70].

**Table 4:** Accuracy of MSK-DUSI for detecting soft-tissue pathology of the shoulder

Target Condition	Study	Reference Standard	Sensitivity	Specificity	LR+	LR-
	Systematic Review					
RC FTT	Lenza 2013 [65]	Arthroscopy or open surgery	0.92	0.93	13.1	0.09
	Smith 2011 [66]	Arthroscopy or open surgery	0.96	0.93	13.7	0.04
	Ottenheijm 2010 [67]	Arthroscopy or MRI	0.95	0.96	23.8	0.05
	Kelly 2009 [64]	MRI	0.87	0.96	21.8	0.14
	de Jesus 2009 [63]	Arthroscopy or open surgery	0.92	0.94	16.5	0.08
	Diagnostic Study					
	Alavekios 2013 [68]	MRI	0.95	0.90	9.50	0.06
	Le Corroller 2008 [69]	MRA	0.91	0.91	10.1	0.10
	Murphey 2013 [49]	Arthroscopy	0.90	0.97	30.0	0.10
	Ok 2013 [70]	Arthroscopy	0.80	0.86	5.71	0.23
	Systematic Review					
RC PTT	Lenza 2013 [65]	Arthroscopy or open surgery	0.52	0.93	7.43	0.52
	Smith 2011 [66]	Arthroscopy or open surgery	0.84	0.89	7.64	0.18
	Ottenheijm 2010 [67]	Arthroscopy or MRI	0.72	0.93	10.3	0.30
	Kelly 2009 [64]	MRI	0.67	0.94	11.2	0.35
	de Jesus 2009 [63]	Arthroscopy or open surgery	0.67	0.94	11.2	0.35
	Diagnostic Study					
	Le Corroller 2008 [69]	MRA	0.71	0.98	35.5	0.30
	Ok 2013 [70]	Arthroscopy	0.46	0.75	1.84	0.72
	Systematic Review					
Bursitis	Ottenheijm 2010 [67]	Arthroscopy or MRI	0.79–0.81	0.94–0.98	12.8–41.5	0.20–0.22
	Diagnostic Study					
	Le Corroller 2008 [69]	MRA	0.96	0.90	9.60	0.04
	Systematic Review					
CT	Ottenheijm 2010 [67]	Arthroscopy or MRI	1.00	0.85–0.98	6.5–51.8	0.02–0.06
	Systematic Review					
RCT	Ottenheijm 2010 [67]	Arthroscopy or MRI	0.67–0.93	0.88–1.00	5.73–41.5	0.07–0.38
	Diagnostic Study					
	Le Corroller 2008 [69]	MRA	0.89	0.96	22.3	0.12
RC atrophy	Khoury 2008 [72]	MRI	0.84	1.00	-	0.16
	Strobel 2005 [75]	MRI	0.78	0.81	4.11	0.27
Subacromial impingement	Read 1998 [73]	Clinical Diagnosis	0.97	0.63	2.62	0.05
LHB						
Full rupture	Armstrong 2006 [71]	Arthroscopy	1.00	1.00	-	-
	Le Corroller 2008 [69]	MRA	0.86	0.98	43.0	0.14
	Read 1998 [73]	Arthroscopy	0.75	1.00	-	0.25
	Skendzel 2011 [74]	Arthroscopy	0.88	0.98	44.0	0.12
Dislocation	Armstrong 2006 [71]	Arthroscopy	0.96	1.00	-	0.04
	Le Corroller 2008 [69]	MRA	0.86	0.98	43.0	0.14
	Read 1998 [73]	Arthroscopy	1.00	1.00	-	-
Tendinitis	Le Corroller 2008 [69]	MRA	0.86	0.98	43.0	0.14
	Read 1998 [73]	Arthroscopy	1.00	1.00	-	-

*MRA* magnetic resonance arthrography; *RC* rotator cuff*; FTT* full tendon tear*; PTT* partial tendon tear*; CT* calcific tendonitis*; RCT* rotator cuff tendinopathy (includes tendinosis and tendinitis)*; LHB* long head of the biceps tendon

The results showed that ultrasound has a high diagnostic value for calcific tendinitis (supraspinatus), full-thickness tears and dislocation of the long head of the biceps [67, 69, 71, 73, 74]. Ultrasound can rule in and out subacromial bursitis with moderate to high accuracy [67, 69], and appeared to be able to rule in rotator cuff tendinopathy accurately, however the SnS results conflicted [67, 69]. One study included in Ottenheijm’s et al. 67] review reported a low SnS (0.67), which was possibly explained by a small population and out-dated ultrasound technology. Ultrasound can rule in rotator cuff atrophy with moderate to high accuracy but is less sensitive in ruling it out [72, 75]. Refer to Table 4 for the individual SnS, SpC and LR outcomes for each of the above conditions. This review found no diagnostic studies assessing the accuracy of ultrasound diagnosis of adhesive capsulitis, pectoralis tears, deltoid tears or partial-tears, tendinosis, calcific tendinitis of the long head of the biceps.

In the shoulder region, the results suggest the use of MSK-DUSI is indicated for any rotator cuff tear, however is less sensitive in ruling out partial-thickness tears. To a lesser extent ultrasound is indicated to diagnose bursitis, calcific tendinitis, rotator cuff tendinopathy, rotator cuff atrophy, subacromial impingement syndrome and long head of the biceps pathology. A summary of recommendations are presented in Table 5. It is important to emphasise that this information is a summary of the results and should be interpreted with consideration of the full results table (Table 4).

**Table 5:** Accuracy Summary – Musculoskeletal Clinical Indications for the use of Diagnostic Ultrasound for the Shoulder Region

Target Condition	Recommendation
Tendons and soft tissue	Grade
Calcific Tendinitis	3
Full thickness rotator cuff tears	3
LHB: dislocation	3
LHB: full thickness tears	3
LHB: tendinitis	3
Rotator cuff tendinopathy	3
Subacromial bursitis	3
Partial thickness rotator cuff tears	2
Rotator cuff atrophy	2
Subacromial Impingement	2
Adhesive capsulitis	Unknown
Deltoid tears	Unknown
LHB: partial thickness tears	Unknown
Pectoralis tears	Unknown

*LHB* long head of biceps

Unknown: No diagnostic accuracy studies found

Grade 0: Not indicated

Grade 1: Conflicting evidence (test results should be interpreted with caution)

Grade 2: Equivalent to other imaging techniques (other techniques might provide significant information)

Grade 3: First choice technique (other techniques rarely provide more information)

### Elbow

A total of 11 clinical conditions were identified (Table 6). Eight diagnostic studies and two systematic reviews relevant to the accuracy of MSK-DUSI for diagnosing soft-tissue pathology of the elbow were found. One systematic review investigated lateral epicondylalgia [76] and one was a systematic review investigating cubital tunnel syndrome [77]. The systematic reviews contained six of the same articles found in this review. These articles were not treated individually in this review as they were included by way of each systematic review (refer to systematic reviews for these references). No other relevant diagnostic studies published after the date of the latest systematic review were found. Two relevant diagnostic studies outside the scope the systematic reviews were found [78, 79]. Therefore, a total of four relevant articles were used in this review (two diagnostic studies and two systematic reviews) [76–79], amounting to 8 individual studies in all. The study characteristics are presented in Table 7.

**Table 6:** Identified clinical conditions of the elbow

Identified clinical conditions of the elbow	Relevant studies found (Yes/No)
Cubital tunnel syndrome	Y
Lateral epicondylalgia (itis/osis)	Y
Medial epicondylalgia (itis/osis)	Y
Biceps tendon injury	Y
Ulnar nerve subluxation	N
Radial nerve compression	N
Median nerve entrapment/pronator syndrome	N
Lateral collateral ligament injury	N
Medial collateral ligament injury	N
Triceps tendon injury	N
Bursitis	N

**Table 7:** Elbow: Study Characteristics

Study	Target Condition	Number of studies (Systematic Review)	Subjects	Mean Age (years)	Mean time from injury to ultrasound	Ultrasound transducer frequency (MHz)	Ultrasound reviewers
Systematic Review							
Beekman et al., 2003 [80]	UNN/CTS	7	542	39.2	N/S	5.0 to 12	RAD and Non-RAD
Dones et al., 2014 [76]	LE	7	211	50	>6 weeks	5.0 to 15	RAD and Non-RAD
Diagnostic Study							
Lobo et al., 2013 [78]	BTI	-	45	44	34.5 days	6.0 to 17.5	RAD
Park et al., 2008 [79]	ME	-	18	50	17.6 months	7.5 to 15	RAD
-	UNS	-	-	-	-	-	-
-	RNC	-	-	-	-	-	-
-	MNE/PS	-	-	-	-	-	-
-	LCL	-	-	-	-	-	-
-	MCL	-	-	-	-	-	-
-	Bursitis	-	-	-	-	-	-
-	TTI	-	-	-	-	-	-

*N/S* not stated; *RAD* Radiologist; *LE* lateral epicondylalgia; *ME* medial epicondylalgia; *BTI* biceps tendon injury; *UNN/CTS* ulnar nerve neuropathy/cubital tunnel syndrome; *UNS* ulnar nerve subluxation; *RNC* radial nerve compression; *MNE/PS* median nerve entrapment/pronator syndrome; *LCL* lateral collateral ligament; *MCL* medial collateral ligament; *TTI* triceps tendon injury

Table 7 reports, the two systematic reviews included 14 diagnostic studies [76, 77]. The included studies reviewed 816 patients [76–79]. The mean age of the cohorts was 45.8 (SD 5.2). Mean time from injury to imaging was not stated in one study [77]. In the three studies where this was stated [76, 78, 79], the time varied from 34.5 days [78] to 17.6 months [79]. All studies documented the job title of the person who performed and reviewed the ultrasound images. In two studies [78, 79], a radiologist performed and interpreted the images and in the remaining two studies [76, 77] a radiologist and non-radiologist were involved. Non-radiologists were either a sonographer or physician [76, 77].

The individual SnS, SpC and LRs for the ultrasound diagnosis of musculoskeletal soft-tissue pathology of the elbow are presented in Table 8. Ulnar nerve thickening at the elbow (the cross-sectional area) is the most common sonographic characteristic used to diagnose cubital tunnel syndrome [77]. Therefore, the results reflect the SnS and SpC of this sonographic characteristic to diagnose cubital tunnel syndrome. One systematic review assessed the accuracy of ultrasound detection for ulnar nerve neuropathy (cubital tunnel syndrome) at the elbow [77]. This review demonstrated that ultrasound can be helpful in the diagnosis of cubital tunnel syndrome, with moderate diagnostic accuracy in demonstrating ulnar nerve thickening and also by detecting underlying abnormalities [77]. One systematic review assessed the accuracy of ultrasound detection for lateral epicondylalgia [76]. This review demonstrated the use of grey-scale ultrasound has moderate diagnostic accuracy in objectively diagnosing lateral epicondylalgia [76].

**Table 8:** Accuracy of MSK-DUSI for detecting soft tissue pathology of the elbow

Target Condition	Study	Reference Standard	Sensitivity	Specificity	LR+	LR-
	Systematic Review					
UNN/CTS	Beekman 2003 [80]	EMG or NCS	0.46–1.00	0.71–0.97	2.88–14.3	0.00–0.64
LE	Dones 2014 [76]	Clinical Diagnosis	0.64	0.82	3.56	0.44
	Diagnostic Study					
ME	Park 2008 [79]	Clinical Diagnosis	0.95	0.92	11.9	0.05
BTI						
Full Rupture	Lobo 2013 [78]	Clinical Diagnosis or open surgery	0.95	0.71	3.28	0.07

*LE* lateral epicondylalgia; *ME* medial epicondylalgia; *BTI* biceps tendon injury; *UNN/CTS* ulnar nerve neuropathy/cubital tunnel syndrome

The results showed that ultrasound has a high diagnostic value for detecting medial epicondylalgia [79] and that ultrasound can rule out full rupture of the distal biceps with high diagnostic accuracy but is only moderately accurate in ruling it in [78]. Refer to Table 8 for the individual SnS, SpC and LR outcomes for each of the above conditions. This review found no diagnostic studies assessing the accuracy of ultrasound diagnosis of partial distal bicep tendon tears, bursitis, lateral or medial collateral ligament injury, triceps tendon injury (tears and snapping triceps syndrome), ulnar nerve subluxation, radial nerve compression or median nerve entrapment/pronator syndrome.

In the elbow region, the results suggest the use of MSK-DUSI is indicated for assisting in the diagnosis of cubital tunnel syndrome and objectively diagnosing lateral epicondylalgia. To a lesser extent, ultrasound is indicated to diagnose medial epicondylalgia and full rupture of the distal biceps tendon. A summary of recommendations are presented in Table 9. It is important to emphasise that this information is a summary of the results and should be interpreted with consideration of the full results table (Table 8).

**Table 9:** Accuracy Summary – Musculoskeletal Clinical Indications for the use of Diagnostic Ultrasound for the Elbow Region

Target Condition	Recommendation
Tendons and soft tissue	
Medial epicondylalgia	3
Lateral epicondylalgia	3
BTI: full thickness tears	2
BTI: partial thickness tears	Unknown
Bursitis	Unknown
LCL and MCL injury	Unknown
Triceps tendon injury	Unknown
Nerves	
Cubital tunnel syndrome	2
Median nerve entrapment	Unknown
Radial nerve compression	Unknown
Ulnar nerve subluxation	Unknown

*BTI* biceps tendon injury; *LCL* lateral collateral ligament; *MCL* medial collateral ligament

Unknown: No diagnostic accuracy studies found

Grade 0: Not indicated

Grade 1: Conflicting evidence (test results should be interpreted with caution)

Grade 2: Equivalent to other imaging techniques (other techniques might provide significant information)

Grade 3: First choice technique (other techniques rarely provide more information)

### Wrist/hand

A total of 10 clinical conditions were identified (Table 10). Sixty-three diagnostic studies and four systematic reviews relevant to the accuracy of MSK-DUSI for diagnosing soft-tissue pathology of the wrist/hand were found. The four systematic reviews investigated idiopathic carpal tunnel syndrome [80–83]. The systematic reviews contained 48 of the same articles found in this review. These articles were not treated individually in this review as they were included by way of each systematic review (refer to systematic reviews for these references). This left five relevant diagnostic studies published after the date of the latest systematic review [84–88] and 10 relevant diagnostic studies outside the scope the systematic reviews [89–98]. Therefore, a total of 19 relevant articles were used in this review (15 diagnostic studies and four systematic reviews) [80–98], amounting to 63 individual studies in all. The study characteristics are presented in Table 11.

**Table 10:** Identified clinical conditions of the wrist/hand

Identified clinical conditions of the wrist/hand	Relevant Studies Found (Yes/No)
Carpal tunnel syndrome	Y
Ligament Injury	Y
de Quervains	Y
Ganglion Cyst	Y
Guyons canal neuropathy	N
Wartenberg syndrome	N
Intersection syndrome	N
Rugby/Jersey finger	N
Trigger finger	N
Tendinopathy (other)	N

**Table 11:** Wrist/Hand: Study Characteristics

Study	Target Condition	Number of studies (Systematic Review)	Subjects	Mean Age (years)	Mean time from injury to ultrasound	Ultrasound transducer frequency (MHz)	Ultrasound reviewers
	Systematic Review						
Cartwright et al., 2012 [81]	CTS	45	1450	N/S	N/S	N/S	RAD and Non-RAD
Descatha et al., 2012 [82]	CTS	13	456	N/S	N/S	3.0 to 13	RAD and Non-RAD
Roll et al., 2011 [83]	CTS	23	890	48	N/S	5.0 to 18	RAD and Non-RAD
Beekman et al., 2003 [80]	CTS	7	268	N/S	N/S	7.0 to 10	RAD and Non-RAD
	Diagnostic Study						
Deniz et al., 2012 [84]	CTS	-	54	46	N/S	10	RAD
Kim et al., 2012 [85]	CTS	-	135	53	N/S	N/S	N/S
Moghtaderi et al., 2012 [86]	CTS	-	79	43	N/S	10 to 13	RAD
Ooi et al., 2014 [87]	CTS	-	51	55	N/S	5.0 to 17	Non-RAD
Tajika et al., 2013 [88]	CTS	-	79	58.6	N/S	6.0 to 14	Non-RAD
Chuter et al., 2009 [90]	Ligament Injury	-	127	40	N/S	N/S	N/S
Dao et al., 2004 [91]	Ligament Injury	-	32	29	N/S	5.0 to 10	RAD
Finlay et al., 2004 [92]	Ligament Injury	-	26	34	N/S	9.0 to 13	RAD
Hergan et al., 1995 [93]	Ligament Injury	-	17	N/S	N/S	N/S	N/S
Melville et al., 2013 [96]	Ligament Injury	-	26	40	33 days	10 to 17	RAD
Taljanovic et al., 2008 [98]	Ligament Injury	-	16	36.4	<12 months	9.0 to 12	RAD
Choi et al., 2011 [89]	DQ	-	13	52.4	19 months	5.0 to 17	RAD
Kwon et al., 2010 [95]	DQ	-	40	51	7.5 months	12 to 15	RAD
Kuwano et al., 2009 [94]	Ganglion	-	183	N/S	N/S	8.5	N/S
Osterwalder et al., 1997 [97]	Ganglion	-	83	N/S	N/S	7.5	RAD
-	Guyons canal	-	-	-	-	-	-
-	WS	-	-	-	-	-	-
-	Rugby/jersey finger	-	-	-	-	-	-
-	Trigger Finger	-	-	-	-	-	-
-	IS	-	-	-	-	-	-
-	T (O)	-	-	-	-	-	-

*N/S* not stated; *RAD* Radiologist; *DQ* de Quervains*; IS* intersection syndrome*; T (O)* tendinopathy (other)*; CTS* carpal tunnel syndrome*; WS* Wartenberg syndrome

Table 11 reports, the four systematic reviews included a total of 88 diagnostic studies [80–83]. The 19 included studies reviewed 4025 patients [80–98]. The mean age was not stated in six studies [80–82, 93, 94, 97]. In the 13 studies where it was stated the mean age of the cohorts was 45.1 (SD 8.9) [83–92, 95, 96, 98]. Mean time from injury to imaging was not stated in 15 studies [80–88, 90–94, 97]. In the four studies where this was stated [89, 95, 96, 98], this ranged from 33 days [96] to 19 months [89]. The ultrasound reviewers were not stated in four studies [85, 90, 93, 94]. In the 15 studies where this was stated; nine studies documented a radiologist performed and interpreted the images [84, 86, 89, 91, 92, 95–98]; four studies documented a radiologist and non-radiologist were involved [80–83]; in the remaining two studies only non-radiologists were involved [87, 88]. Non-radiologists were either a sonographer or physician [80–83, 87, 88].

The individual SnS, SpC and LRs for the ultrasound diagnosis of musculoskeletal soft-tissue pathology of the wrist/hand are presented in Table 12. The quantitative measure commonly reported to support the diagnosis of idiopathic carpal tunnel syndrome was median nerve thickening at the wrist (cross-sectional area) [80]. Therefore, the results reflect the SnS and SpC of this sonographic characteristic to diagnose carpal tunnel syndrome. The four reviews demonstrate that ultrasound has low to moderate diagnostic value in detecting idiopathic carpal tunnel syndrome and had the potential to be used as a screening tool or as a complementary examination to electrodiagnostic studies, however not as an isolated alternative [80–83]. The five diagnostic studies dated after the systematic reviews reported ultrasound has a moderate to high diagnostic value in the detection of carpal tunnel syndrome [84–88]. The presence of discordance between the results of the systematic reviews and diagnostic studies may be the result of severity of disease, operator-interpreter experience, quality of ultrasound equipment and the cut-off measurement used to determine median nerve thickening. Currently, ultrasound scanning technique and measurements for median nerve thickening are not fully standardised [82].

**Table 12:** Accuracy of MSK-DUSI for detecting soft tissue pathology of the wrist/hand

Target Condition	Study	Reference Standard	Sensitivity	Specificity	LR+	LR-
	Systematic Review					
CTS	Cartwright 2012 [81]	Clinical and NCS	0.65–1.00	0.50–0.98	1.66–48.5	0.00–0.38
	Descatha 2012 [82]	Clinical and NCS	0.84	0.78	3.82	0.21
	Roll 2011 [83]	Clinical and NCS	0.29 to 1.00	0.47 to 1.00	1.89–∞	0.00–0.71
	Beekman 2003 [80]	NCS	0.70–0.88	0.57–0.96	1.70–27.3	0.13–0.48
	Diagnostic Study					
	Deniz 2012 [84]	Clinical and NCS	0.84	0.79	4.00	0.20
	Kim 2012 [85]	Clinical and NCS	0.89	0.90	8.90	0.12
	Moghtaderi 2012 [86]	Clinical and NCS	0.83	0.91	9.22	0.19
	Ooi 2014 [87]	NCS	0.92	0.90	9.20	0.09
	Tajika 2013 [88]	NCS	1.00	0.99	100	-
Ligament Injury						
UCL (displaced)	Chuter 2009 [90]	Surgical Findings	0.92	N/S	-	-
	Hergan 1995 [93]	MRI	0.88	0.83	5.18	0.15
	Melville 2013 [96]	Surgical Findings	1.00	1.00	-	-
UCL (non-displaced)	Hergan 1995 [93]	MRI	0.88	0.91	9.78	0.13
	Melville 2013 [96]	Surgical Findings	1.00	1.00	-	-
Scapholunate	Dao 2004 [91]	Arthroscopy	0.46	1.00	-	0.54
	Finley 2004 [92]	Arthrography	1.00	1.00	-	-
	Taljanovic 2008 [98]	MRA	1.00	0.92	12.5	-
Lunotriquetral	Finley 2004 [92]	Arthrography	0.25	1.00	-	0.75
	Taljanovic 2008 [98]	MRA	0.50	0.90	5.00	0.56
TFCC	Finley 2004 [92]	Arthrography	0.64	1.00	-	0.36
	Taljanovic 2008 [98]	MRA	0.86	1.00	-	0.14
de Quervains	Choi 2011 [89]	Surgical Findings	1.00	N/S	-	-
	Kwon 2010 [95]	Surgical Findings	1.00	0.96	25.0	-
Ganglion Cyst	Kuwano 2010 [94]	Surgical Findings	0.39	1.00	-	0.61
	Osterwalder 1997 [97]	Histology and surgical findings	0.93	0.86	6.64	0.08

*UCL* ulnar collateral ligament; *TFCC* triangular fibrocartilage complex; *CTS* carpal tunnel syndrome

The results showed that ultrasound had high diagnostic value for ulnar collateral ligament (UCL) injury (displaced and non-displaced) [90, 93, 96] and high diagnostic value for ruling in triangular fibrocartilage complex (TFCC) injury, but is less sensitive at ruling it out [92, 98]. The results showed ultrasound had a high accuracy in ruling in scapholunate ligament (SLL) and lunotriquetral ligament (LTL) injury (SpC: >0.90), but there was conflicting SnS for SLL injury and low SnS for LTL injury (<0.50) [91, 92, 98]. This indicates that ultrasound may be no better than chance in excluding injury to the LTL. Dao et al. [91] reported a low SnS (0.46) of ultrasound for detecting SLL injury and although the methodological quality the study was strong it might be explained by the small sample size and difficulty in reproducing dynamic manoeuvres . Refer to Table 12 for the individual SnS, SpC and LR values for the above conditions. This review found no diagnostic studies assessing the accuracy of ultrasound for detecting Guyons canal neuropathy, Wartenberg syndrome, Intersection syndrome, rugby/jersey finger, trigger finger or other tendinopathy.

In the wrist/hand region, the results suggest that MSK-DUSI has moderate diagnostic value for detecting idiopathic carpal tunnel syndrome and is indicated as a screening tool or complementary test to electrodiagnostic studies. To a lesser extent ultrasound is indicated to: rule in and out displaced and non-displaced ulnar collateral ligament tears and de Quervains; rule in ganglions cysts and scapholunate ligament tears, however conflicting results are present for the ability of ultrasound to rule them out; rule in TFCC injury and lunotriquetral ligament tears but not to rule them out. A summary of recommendations are presented in Table 13. It is important to emphasise that this information is a summary of the results and should be interpreted with consideration of the full results table (Table 12).

**Table 13:** Accuracy Summary – Musculoskeletal Clinical Indications for the use of Diagnostic Ultrasound for the Wrist/Hand Region

Target Condition	Recommendation
Tendons and soft tissue	Grade
de Quervains	3
Ganglion cyst	3
Lunotriquetral ligament injury	2
Ulnar collateral ligament (displaced)	2
Ulnar collateral ligament (non-displaced)	2
Scapholunate ligament injury	1
TFCC injury	1
Intersection syndrome	Unknown
Rugby/jersey finger	Unknown
Trigger finger	Unknown
Other tendinopathy	Unknown
Nerves	
Carpal tunnel syndrome	2
Guyons canal neuropathy	Unknown
Wartenberg syndrome	Unknown

*TFCC* triangular fibrocartilage complex

Unknown: No diagnostic accuracy studies found

Grade 0: Not indicated

Grade 1: Conflicting evidence (test results should be interpreted with caution)

Grade 2: Equivalent to other imaging techniques (other techniques might provide significant information)

Grade 3: First choice technique (other techniques rarely provide more information)

### Hip

A total of 6 clinical conditions were identified (Table 14). Eight diagnostic studies and one systematic review relevant to the accuracy of MSK-DUSI for diagnosing soft-tissue pathology of the hip were found. The systematic review investigated gluteal tendon tears [99]. The systematic review contained seven of the same articles found in this review. These articles were not treated individually in this review as they were included by way of the systematic review (refer to systematic reviews for these references). No other relevant diagnostic studies published after the date of the latest systematic review were found. Two relevant diagnostic studies outside the scope the systematic reviews were found [100, 101]. Therefore, a total of three relevant articles were used in this review (two diagnostic studies and one systematic review) [99–101], amounting to nine individual studies in all. The study characteristics are presented in Table 15.

**Table 14:** Identified clinical conditions of the hip

Identified clinical conditions of the hip	Relevant Studies Found (Yes/No)
Muscle/tendon injury (gluteal, psoas, hamstrings, quadriceps)	Y
Bursitis (trochanteric, iliopsoas)	Y
Meralgia paresthetica	Y
Snapping hip syndrome (extra-articular)	N
Sciatica	N
Femoral nerve injury	N

**Table 15:** Hip: Study Characteristics

Study	Target Condition	Number of studies (Systematic Review)	Subjects	Mean Age (years)	Mean time from injury to ultrasound	Ultrasound transducer frequency (MHz)	Ultrasound reviewers
Systematic Review							
Westacott et al., 2011 [99]	Muscle/Tendon Injury	7	N/S	N/S	N/S	7.5 to 18	RAD
Diagnostic Study							
Fearon et al., 2010 [100]	Bursitis	-	24	56	33.8 months	7.0	N/S
Suh et al., 2013 [101]	LFC/MP	-	23	46	4 months	5.0 to 12	Non-RAD
-	Sciatica	-	-	-	-	-	-
-	Femoral Nerve	-	-	-	-	-	-
-	Snapping hip (E)	-	-	-	-	-	-

*N/S* not stated; *RAD* Radiologist; *LFT/MP* lateral femoral cutaneous/meralgia paresthetica; *(E) (*extra-articular)

Table 15 reports, the one systematic review included seven diagnostic studies [99]. One study did not state the number of subjects [99]. In the two studies where this was stated the studies reviewed 47 patients [100, 101]. The mean age was not stated in one study [99]. In the two studies where this was stated the mean age of the cohorts was 51 (SD 7.1) [100, 101]. Mean time from injury to imaging was not stated in one study [99]. In the two studies where this was stated [100, 101], this ranged from 4 months [101] to 33.8 months [100]. One study did not report on who performed and reviewed the ultrasound images [100]. In the two studies where this was reported, one study recorded that a radiologist performed and interpreted the images [99] and in the remaining study a sonographer performed and interpreted the images [101].

The individual SnS, SpC and LRs for the ultrasound diagnosis of musculoskeletal soft-tissue pathology of the hip are presented in Table 16. The results of the systematic review demonstrate that ultrasound has moderate to high diagnostic accuracy for detecting any tear of the gluteal tendon and may prove to be the investigation of choice [99]. The results show that ultrasound has a moderate diagnostic value for ruling out trochanteric bursitis, but has high diagnostic value of ruling it in [100]. For meralgia paresthetica the results show that ultrasound has a high diagnostic value for detecting meralgia paresthetica [101]. This review found no diagnostic studies assessing the accuracy of ultrasound diagnosis muscle or tendon injury of the psoas, hamstrings and quadriceps; iliopsoas bursitis, snapping hip syndrome (extra-articular), sciatica, or femoral nerve injury.

**Table 16:** Accuracy of MSK-DUSI for detecting soft tissue pathology of the hip

Target Condition	Study	Reference Standard	Sensitivity	Specificity	LR+	LR-
	Systematic Review					
Gluteal Tendon Tear	Westacott 2011 [99]	MRI	0.79–1.00	0.95–1.00	15.8–∞	0.00–0.21
	Diagnostic Study					
Trochanteric bursitis	Fearon 2010 [100]	Surgical findings and histology	0.69	1.00	-	0.31
LFC/MP	Suh 2013 [101]	Clinical and NCS	0.96	0.96	24.0	0.04

*LFT/MP* lateral femoral cutaneous/meralgia paresthetica

In the hip region, the results suggest the use of MSK-DUSI is indicated for any gluteal tendon tear due to its moderate to high diagnostic accuracy and to a lesser extent for the diagnosis of trochanteric bursitis and meralgia paresthetica. A summary of recommendations are presented in Table 17. It is important to emphasise that this information is a summary of the results and should be interpreted with consideration of the full results table (Table 16).

**Table 17:** Accuracy Summary – Musculoskeletal Clinical Indications for the use of Diagnostic Ultrasound for the Hip Region

Target Condition	Recommendation
Tendons and soft tissue	Grade
Gluteal tendon tears	3
Trochanteric bursitis	2
Iliopsoas bursitis	Unknown
Psoas/hamstring/quadriceps injury	Unknown
Snapping hip syndrome (extra-articular)	Unknown
Nerves	
Meralgia paresthetica	3
Femoral nerve injury	Unknown
Sciatica (causes)	Unknown

Unknown: No diagnostic accuracy studies found

Grade 0: Not indicated

Grade 1: Conflicting evidence (test results should be interpreted with caution)

Grade 2: Equivalent to other imaging techniques (other techniques might provide significant information)

Grade 3: First choice technique (other techniques rarely provide more information)

### Knee

A total of 19 clinical conditions were identified (Table 18). Twenty diagnostic studies pertaining to the accuracy of MSK-DUSI for diagnosing musculoskeletal soft-tissue pathology of the knee were found [102–121]. No systematic reviews were found. The study characteristics are presented in Table 19.

**Table 18:** Identified clinical conditions of the knee

Identified clinical conditions of the knee	Relevant studies found (Yes/No)
Quadriceps tendon injury	Y
Patella tendon injury	Y
Popliteus tendon injury	Y
Anterior cruciate ligament injury	Y
Lateral collateral ligament injury	Y
Plica syndrome	Y
Baker’s cyst	Y
Meniscal tears	Y
Meniscal cyst	Y
Common peroneal neuropathy	Y
Bursitis	N
Ganglion	N
Iliotibial band friction syndrome	N
Hoffer’s fat pad syndrome	N
Retinacula pathology	N
Medial collateral ligament injury	N
Posterior cruciate ligament injury	N
Hamstring injury	N
Gastrocnemius injury	N

**Table 19:** Knee: Study Characteristics

Study	Target Condition	Number of studies (Systematic Review)	Subjects	Mean Age (years)	Mean time from injury to ultrasound	Ultrasound transducer frequency (MHz)	Ultrasound reviewers
Diagnostic Study							
Bianchi et al., 1994 [104]	Tendinopathy/Tear	-	29	41	N/S	7.5	RAD
Garrick et al., 2008 [107]	Tendinopathy/Tear	-	63	N/S	N/S	10 to 14	RAD
Sekiya et al., 2010 [114]	Tendinopathy/Tear; Ligament Injury	-	16	N/S	N/S	10 to 14	RAD
Warden et al., 2007 [120]	Tendinopathy/Tear	-	30	27	N/S	10 to 14	RAD
Ward et al., 2001 [119]	Baker’s Cyst	-	36	46	78 days	7.0 to 10	RAD
Derks et al., 1986 [105]	Plica syndrome	-	38	N/S	N/S	7.5	RAD
Paczesny et al., 2009 [110]	Plica Syndrome	-	88	20	6 months	12	Non-RAD
Fuchs et al., 2002 [106]	Ligament Injury	-	193	N/S	N/S	10 to 14	RAD
Khan et al., 2006 [108]	Ligament Injury; Meniscal Tear	-	60	35	N/S	7.5	RAD
Ptasznik et al., 1995 [112]	Ligament Injury	-	37	27	3.3 weeks	7.5	RAD
Skovgaard et al., 2000 [116]	Ligament Injury	-	62	29.2	9 days	7.0	RAD
Alizadeh et al., 2013 [102]	Meniscal Tear	-	37	43.5	N/S	14	RAD
Azzoni et al., 2002 [103]	Meniscal Tear	-	216	27.5	N/S	7.5 to 10	RAD
Najafi et al., 2006 [109]	Meniscal Tear	-	100	N/S	N/S	6.5	RAD
Park et al., 2008 [79]	Meniscal Tear	-	22	50.4	N/S	7.5 to 15	RAD
Shetty et al., 2008 [115]	Meniscal Tear	-	35	47	N/S	5.0 to 13	RAD
Wareluk et al., 2012 [121]	Meniscal Tear	-	80	36.2	<1 month	6.0 to 12	Non-RAD
Rutten et al., 1998 [113]	Meniscal cyst/Meniscal Tear	-	50	51	N/S	7.5	N/S
Sorrentino et al., 2007 [117]	Meniscal cyst	-	104	43	N/S	7 to 12	RAD
Visser et al., 2013 [118]	Nerve Injury	-	103	53	5 weeks	7 to 18	N/S
-	Bursitis	-	-	-	-	-	-
-	Ganglion	-	-	-	-	-	-
-	ITB friction	-	-	-	-	-	-
-	HFPS	-	-	-	-	-	-
-	Retinacula	-	-	-	-	-	-

*N/S* not stated; *RAD* Radiologist; *ITB* iliotibial band*; HFPS* Hoffer’s fat pad syndrome

Table 19 reports, the 20 included studies reviewed 1399 patients [102–121]. The mean age was not stated in five studies [105–107, 109, 114]. In the 15 studies where it was stated the mean age of the cohorts was 38.5 (SD 10.4) [102–104, 108, 110–113, 115–121]. Mean time from injury to imaging was not stated in 14 studies [102–104, 106–109, 111, 113–115, 117, 119, 120]. In the six studies where this was stated [110, 112, 116, 118, 119, 121], it ranged from 3.3 days [112] to 6 months [110]. Two studies did not report on who performed and reviewed the ultrasound images [113, 118]. In the 18 studies where this was reported, 16 studies recorded a radiologist performed and interpreted the images [102–109, 111, 112, 114–117, 119, 120] and in two studies a non-radiologist performed and interpreted the images [110, 121]. The non-radiologists were a sonographer and a physician [110, 121].

The individual SnS, SpC and LRs for the ultrasound diagnosis of musculoskeletal soft-tissue pathology of the knee are presented in Table 20. The results show that ultrasound has a moderate to high diagnostic value for medial and lateral meniscal tears [102, 103, 108, 109, 111, 113, 115, 121]. The results show that ultrasound has a high diagnostic value for full quadriceps tendons tears [104], moderate for patella tendinopathy [107, 120] and injury to the popliteal tendon [114].. Five studies assessed the accuracy of ultrasound detection of ligamentous injury [106, 108, 112, 114, 116]. The results show that ultrasound has moderate to high diagnostic value for full anterior cruciate ligament tears [106, 108, 112, 116] and high diagnostic value at ruling in lateral collateral ligament tears, but low diagnostic value in ruling them out [114]. Ultrasound had a 100 % false negative rate for detecting partial anterior cruciate ligament tears [108]. The results show that ultrasound has high diagnostic value for Baker’s cysts [119] and meniscal cysts [113, 117], with moderate to high diagnostic value for medial-patella plica syndrome [105, 110]. Ultrasound can rule out common peroneal nerve neuropathy with high accuracy, but in less specific at ruling it in [118]. This review found no diagnostic studies assessing the accuracy of ultrasound for detecting muscle or tendon pathology of the hamstrings, iliotibial band and gastrocnemius; pes anserinus tendinobursitis, medial collateral ligament or posterior cruciate ligament injury, ganglions, retinacula pathology or Hoffa’s fat pad syndrome.

**Table 20:** Accuracy of MSK-DUSI for detecting soft tissue pathology of the knee

Target Condition	Study	Reference Standard	Sensitivity	Specificity	LR+	LR-
Tendinopathy/Tear	Diagnostic Study					
Full QTT	Bianchi 1994 [104]	Surgical Findings	1.00	1.00	-	-
Patella Tendinopathy	Garrick 2008 [107]	Clinical and MRI	0.87	0.79	4.14	0.16
	Warden 2007 [120]	Clinical and MRI	0.87	0.82	4.83	0.16
Popliteus Tear	Sekiya 2010 [114]	Surgical Findings	0.67	0.75	2.68	0.44
Ligament Injury						
Full ACL Tear	Fuchs 2002 [106]	MRI	0.91	0.80	4.55	0.11
	Khan 2006 [108]	MRI and Arthroscopy	0.79	1.00	-	0.21
	Ptasznik 1995 [112]	Arthroscopy	0.91	1.00	-	0.09
	Skovgaard 2000 [116]	Arthroscopy	0.88	0.98	-	-
Partial ACL Tear	Khan 2006 [108]	MRI and Arthroscopy	0.00	0.00	-	-
LCL	Sekiya 2010 [114]	Surgical Findings	0.33	1.00	-.	0.67
Plica Syndrome	Derks 1986 [105]	Arthroscopy	0.92	0.73	3.41	0.11
	Paczesny 2009 [110]	Arthroscopy	0.90	0.83	5.29	0.12
Baker’s Cyst	Ward 2001 [119]	MRI	1.00	1.00	-	-
Meniscal Tear						
Medial Meniscus	Alizadeh 2013 [102]	MRI	0.83	0.71	2.86	0.24
	Azzoni 2002 [103]	MRI	0.60	0.21	0.76	1.90
	Khan 2006 [108]	MRI and Arthroscopy	0.93	0.93	13.3	0.08
	Najafi 2006 [109]	Arthroscopy	1.00	0.95	20	0.00
	Park 2008 [79]	MRI	0.86	0.85	5.73	0.16
	Rutten 1998 [113]	Surgical Findings	0.82	0.75	3.29	0.24
	Shetty 2008 [115]	MRI	0.86	0.69	2.77	0.20
	Wareluk 2012 [121]	Arthroscopy	0.93	0.73	3.44	0.10
Lateral Meniscus	Khan 2006 [108]	MRI and Arthroscopy	0.88	1.00	-	0.12
	Najafi 2006 [109]	Arthroscopy	0.93	1.00	-	0.07
	Park 2008 [79]	MRI	0.86	0.85	5.73	0.16
	Rutten 1998 [113]	Surgical Findings	0.82	0.75	3.29	0.24
	Wareluk 2012 [121]	Arthroscopy	0.67	0.96	16.8	0.34
Meniscal Cyst	Rutten 1998 [113]	Surgical Findings	0.97	0.86	6.93	0.03
	Sorrentino 2007 [117]	MRI	0.94	1.00	-	0.06
Nerve Injury						
Common Peroneal Neuropathy	Visser 2013 [118]	NCS	0.90	0.69	2.90	0.14

*QTT* quadriceps tendon tear; *ACL* anterior cruciate ligament; *LCL* lateral collateral ligament

In the knee region, the results suggest MSK-DUSI may be indicated as a screening tool for medial and lateral meniscus tears due to its moderate to high diagnostic accuracy. To a lesser extent ultrasound may be used for the diagnosis of full-thickness quadriceps tendon tears, patella tendinopathy, full-thickness anterior cruciate ligament tears, medial patella plica syndrome, Baker’s cysts and meniscal cysts. Ultrasound can rule in lateral collateral ligament and popliteus tears but is less sensitive at ruling it out and can rule out common peroneal nerve neuropathy but is less sensitive at ruling it in. Ultrasound is not indicated for partial-thickness anterior cruciate ligament tears. A summary of recommendations are presented in Table 21. It is important to emphasise that this information is a summary of the results and should be interpreted with consideration of the full results table (Table 20).

**Table 21:** Accuracy Summary – Musculoskeletal Clinical Indication for Diagnostic Ultrasound of the Knee Region

Target Condition	Recommendation
Tendons and soft tissue	Grade
Full thickness quadriceps tendon tears	3
Patella tendinopathy	3
Baker’s Cyst	2
Medial patella plica syndrome	2
Meniscal cyst	2
Ganglion cyst	Unknown
Hamstring/ITB/gastrocnemius injury	Unknown
Hoffa’s fat pad syndrome	Unknown
Pes anserinus tendinobursitis	Unknown
Internal knee derangement and associated injury	
Full ACL tears	0^a^
Partial ACL tears	0
Medial meniscus tears	0^a^
Lateral meniscus tears	0^a^
LCL injury	0^a^
Popliteal injury	0^a^
MCL injury	Unknown
PCL injury	Unknown
Nerves	
Common peroneal neuropathy	2

*ACL* anterior cruciate ligament; *PCL* posterior cruciate ligament; *LCL* lateral collateral ligament; *MCL* medial collateral ligament; *ITB* iliotibial band

Unknown: No diagnostic accuracy studies found

Grade 0^a^: Not indicated as a definitive diagnostic tool for ligamentous and meniscal tears of the knee, however may have a role as an on field, point-of-care screening tool

Grade 0: Not indicated

Grade 1: Conflicting evidence (test results should be interpreted with caution)

Grade 2: Equivalent to other imaging techniques (other techniques might provide significant information)

Grade 3: First choice technique (other techniques rarely provide more information)

### Ankle/foot

A total of 20 clinical conditions were identified (Table 22). Thirty-five diagnostic articles relevant to the accuracy of MSK-DUSI for diagnosing soft-tissue pathology of the ankle/foot were found [122–156]. No systematic reviews were found. The study characteristics are presented in Table 12.

**Table 22:** Identified clinical conditions of the ankle/foot

Identified clinical conditions of the ankle/foot	Relevant studies found (Yes/No)
Plantaris tendon injury	Y
Plantar plate injury	Y
Peroneal tendon injury	Y
Achilles tendon injury	Y
Posterior tibial tendon injury	Y
Anterior talofibular ligament injury	Y
Posterior talofibular ligament injury	Y
Calcaneofibular ligament injury	Y
Deltoid ligament injury	Y
Syndesmotic injury	Y
Morton’s neuroma	Y
Anterolateral impingement	Y
Peroneal subluxation	Y
Plantar fasciitis	Y
Tarsal tunnel syndrome	N
Bursitis	N
Retinacula pathology	N
Ganglion	N
Tibialis anterior injury	N
Gastrocnemius tears	N

Table 23 reports, the 35 included studies reviewed 1713 patients [122–156]. The mean age was not stated in nine studies [133, 137–139, 141, 150, 153, 155, 156]. In the 26 studies where it was stated the mean age of the cohorts was 42.7 (SD 9.9) [122–132, 134–136, 140, 142–149, 151, 152, 154]. Mean time from injury to imaging was not stated in 24 studies [123, 125, 126, 128, 130, 131, 133–140, 144, 145, 147–151, 153, 154, 156]. In the 11 studies where this was stated [122, 124, 127, 129, 132, 141–143, 146, 152, 155], this ranged from <2 days [132] to 14 months [142]. Two studies did not report on who performed and reviewed the ultrasound images [134, 148]. In the 34 studies where this was reported, 24 studies recorded a radiologist performed and interpreted the images [122, 124–127, 131, 133, 135–137, 140–143, 147, 149–156]; in two studies a radiologist and non-radiologist were involved [123, 128]; and in eight studies only a non-radiologist was involved [129, 130, 132, 138, 139, 144–146]. The non-radiologists consisted of either a sonographer or a physician [123, 128–130, 132, 139, 144–146].

**Table 23:** Ankle/Foot: Study Characteristics

Study	Target Condition	Number of studies (Systematic Review)	Subjects	Mean Age (years)	Mean time from injury to ultrasound	Ultrasound transducer frequency (MHz)	Ultrasound reviewers
Diagnostic Study							
Bianchi et al., 2011 [122]	Tendinopathy/Tear	-	5	47.2	8 days	12.5 to 17.5	RAD
Carlson et al., 2013 [123]	Tendinopathy/Tear	-	8	51.9	N/S	N/S	RAD and Non-RAD
Grant et al., 2005 [127]	Tendinopathy/Tear	-	58	45.2	11.2 months	11 to 15	RAD
Gregg et al., 2006 [128]	Tendinopathy/Tear	-	52	57	N/S	11	RAD and Non-RAD
Hartgerink et al., 2001 [131]	Tendinopathy/Tear	-	26	40	N/S	7.5 to 12	RAD
Kainberger et al., 1990 [134]	Tendinopathy/Tear	-	73	38	N/S	5.0 to 10	N/S
Kalebo et al., 1992 [135]	Tendinopathy/Tear	-	37	35	N/S	7.5	RAD
Kayser et al., 2005 [137]	Tendinopathy/Tear	-	13	N/S	N/S	7.5	RAD
Klein et al., 2012 [138]	Tendinopathy/Tear	-	42	N/S	N/S	15 to 16	Non-RAD
Klein et al., 2013 [139]	Tendinopathy/Tear	-	50	N/S	N/S	15 to 16	Non-RAD
Nallamshetty et al., 2005 [144]	Tendinopathy/Tear	-	18	61	N/S	10	Non-RAD
Premkumar et al., 2002 [149]	Tendinopathy/Tear	-	31	43	N/S	10	RAD
Rockett et al., 1998 [150]	Tendinopathy/Tear	-	28	N/S	N/S	7.5 to 10	RAD
Waitches et al., 1998 [156]	Tendinopathy/Tear	-	33	N/S	N/S	7.5 to10	RAD
Cheng et al., 2014 [124]	Ligament Injury	-	120	32	2.2 years	5.0 to 17	RAD
Guillodo et al., 2010 [129]	Ligament Injury	-	56	30.1	7.6 months	5.0 to 12	Non-RAD
Gun et al., 2013 [130]	Ligament Injury	-	65	34	N/S	7.5	Non-RAD
Henari et al., 2011 [132]	Ligament Injury	-	12	41	<2 days	N/S	Non-RAD
Hua et al., 2012 [133]	Ligament Injury	-	83	N/S	N/S	7.5	RAD
Margetic et al., 2012 [141]	Ligament Injury	-	30	N/S	1 week	7.0 to 15	RAD
Mei-Dan et al., 2009 [143]	Ligament Injury	-	47	27	12 days	7.5 to 12	RAD
Oae et al., 2010 [146]	Ligament Injury	-	34	29	1 week	9.0	Non-RAD
van Dijk et al., 1996 [155]	Ligament Injury	-	160	N/S	<1 week	N/S	RAD
Fazal et al., 2012 [126]	Morton’s Neuroma	-	47	46	N/S	5.0 t o12	RAD
Kankanala et al., 2007 [136]	Morton’s Neuroma	-	48	52.6	N/S	13.5	RAD
Lee et al., 2007 [140]	Morton’s Neuroma	-	17	48.6	N/S	9.0	RAD
Oliver et al., 1998 [147]	Morton’s Neuroma	-	37	49.6	N/S	7.5	RAD
Pastides et al., 2012 [148]	Morton’s Neuroma	-	36	43.8	N/S	N/S	N/S
Sharp et al., 2003 [152]	Morton’s neuroma	-	25	52	8 months	12	RAD
Sobiesk et al., 1997 [153]	Morton’s Neuroma	-	20	N/S	N/S	7.5	RAD
Torres-Claramunt et al., 2012 [154]	Morton’s Neuroma	-	37	60.6	N/S	7.5 to 9.0	RAD
Cochet et al., 2010 [125]	Impingement	-	41	32	N/S	5.0 to 12	RAD
McCarthy et al., 2008 [142]	Impingement	-	17	32	14 months	5.0 to 12	RAD
Neustadter et al., 2004 [145]	Peroneal Subluxation	-	13	30.4	N/S	12 to 13	Non-RAD
Sabir et al., 2005 [151]	Plantar Fasciitis	-	77	45.9	N/S	6.0 to 9.0	RAD
-	TTS	-	-	-	-	-	-
-	Bursitis	-	-	-	-	-	-
-	Retinacula	-	-	-	-	-	-
-	Ganglion	-	-	-	-	-	-

*N/S* not stated; *RAD* Radiologist; *TTS* tarsal tunnel syndrome

The individual SnS, SpC and LRs for the ultrasound diagnosis of musculoskeletal soft-tissue pathology of the ankle/foot are presented in Table 24. The results show that ultrasound has high diagnostic value for peroneal subluxation [145], anterior talofibular [124, 129, 130, 133, 141, 146, 155], posterior talofibular [124], calcaneofibular [124, 141], deltoid [132] and syndesmotic ligament injury [143]. The high accuracy of posterior talofibular ligament injury was based off one subject, therefore the use of ultrasound for this condition is not recommended due to lack of evidence. Ultrasound has high diagnostic accuracy for ruling in Morton’s neuroma, but is less sensitive at ruling it out [126, 136, 140, 147, 148, 152–154].

**Table 24:** Accuracy of MSK-DUSI for detecting soft tissue pathology of the ankle/foot

Target Condition	Study	Reference Standard	Sensitivity	Specificity	LR+	LR-
	Diagnostic Study					
Tendinopathy/Tear						
Plantaris Tendon Tear	Bianchi 2011 [122]	MRI	1.00	1.00	-	-
Plantar Plate Tear	Carlson 2013 [123]	Surgical Findings	1.00	0.60	2.50	-
	Gregg 2006 [128]	MRI	0.86	0.64	2.39	0.22
	Klein 2013 [139]	MRI	0.91	0.25	1.21	0.36
	Klein 2012 [138]	MRI	0.92	0.25	1.23	0.32
Peroneal Tendon Tear	Grant 2005 [127]	Surgical Findings	1.00	0.85	6.67	-
	Waitches 1998 [156]	Surgical Findings	1.00	0.79	4.76	-
Achilles Tendinopathy	Hartgerink 2001 [131]	Surgical Findings	1.00	0.83	5.88	-
	Kainberger 1990 [134]	Clinical and MRI	0.72	0.83	4.24	0.34
	Kalebo 1992 [135]	Surgical Findings	0.94	1.00	-	0.06
	Kayser 2005 [137]	MRI	0.50	0.81	2.63	0.62
Posterior Tibial Tendinopathy	Nallamshetty 2005 [144]	MRI	0.78	1.00	-	0.22
	Premkumar 2002 [149]	MRI	0.80	0.90	8.00	0.22
	Rockett 1998 [150]	Surgical Findings	1.00	0.90	10.0	-
	Waitches 1998 [156]	Surgical Findings	1.00	1.00	-	-
Ligament Injury						
ATF	Cheng 2014 [124]	Surgical Findings	0.99	0.96	24.8	0.01
	Guillodo 2010 [129]	Arthrography	0.85	1.00	-	0.15
	Gun 2013 [130]	MRI	0.94	1.00	-	0.06
	Hua 2012 [133]	Surgical Findings	0.98	0.92	12.3	0.02
	Margetic 2012 [141]	MRI	1.00	1.00	-	-
	Oae 2010 [146]	Surgical Findings	1.00	0.33	1.49	-
	van Dijk 1996 [155]	Arthrography	0.92	0.64	2.56	0.13
PTF	Cheng 2014 [124]	Surgical Findings	1.00	1.00	-	-
CF	Cheng 2014 [124]	Surgical Findings	0.94	0.91	10.4	0.07
	Margetic 2012 [141]	MRI	1.00	1.00	-	-
Deltoid	Henari 2011 [132]	Arthrography	1.00	1.00	-	-
Syndesmotic	Mei-Dan 2009 [143]	MRI	1.00	1.00	-	-
Morton’s Neuroma	Fazal 2012 [126]	Surgical Findings	0.96	1.00	-	0.04
	Kankanla 2007 [136]	Surgical and Histology	0.91	1.00	-	0.09
	Lee 2007 [140]	Surgical Findings	0.79	1.00	-	0.21
	Oliver 1998 [147]	Surgical and Histology	0.96	1.00	-	0.04
	Pastides 2012 [148]	Surgical Findings	0.90	1.00	-	0.10
	Sharp 2003 [152]	Surgical and Histology	0.79	1.00	-	0.21
	Sobiesk 1997 [153]	Surgical Findings	1.00	0.83	5.88	-
	Torres-Claramunt 2012 [154]	Surgical and Histology	0.57	1.00	-	0.43
Anterolateral Impingement	Cochet 2010 [125]	Arthrography	0.77	0.57	1.79	0.40
	McCarthy 2008 [142]	Surgical Findings	1.00	1.00	-	-
Peroneal Subluxation	Neustadter 2004 [145]	Surgical Findings	1.00	1.00	-	-
Plantar Fasciitis	Sabir 2005 [151]	MRI	0.80	0.89	7.27	0.22

*ATF* anterior talofibular; *PTF* posterior talofibular; *CF* calcaneofibular

The results show that ultrasound has high diagnostic value for plantaris tendon tears [122]; moderate to high for peroneal tendon tears [127, 156], Achilles tendinopathy [131, 134, 135, 137] and posterior tibial tendinopathy [144, 149, 150, 156]; moderate for plantar fasciitis [151]. Ultrasound can rule out plantar plate tears with high accuracy, but has low accuracy when ruling them in [123, 128, 138, 139]. The low SpC significantly reduces the overall accuracy of ultrasound for this condition. Two studies assessed the accuracy of ultrasound detection of anterolateral ankle impingement, reporting significant differences in SnS and SpC [125, 142]. The difference in diagnostic accuracy is likely due to the heterogenic study population and study size. Cochet et al. [125] report on 41 subjects from the general population whereas McCarthy et al. [142] reported on 17 subject from a population of elite athletes. This review found no diagnostic studies assessing the accuracy of ultrasound diagnosis for tibialis anterior tendinopathy, gastrocnemius tears, bursitis, retinaculum pathology, ganglion or tarsal tunnel syndrome.

In the ankle/foot region, the results suggest the use of MSK-DUSI is indicated for anterior talofibular ligament injury and Morton’s neuroma due to its high diagnostic accuracy. To a lesser extent ultrasound may be used for the diagnosis of plantaris tendon tears, peroneal tendon tears, posterior tibial tendinopathy; calcaneofibular ligament, deltoid ligament and syndesmotic injury; peroneal subluxation and plantar fasciitis. There is conflicting evidence to indicate ultrasound for the detection of Achilles tendon tendinopathy and anterolateral ankle impingement. Ultrasound can rule out plantar plate tears but it is less sensitive at ruling them in. Ultrasound is not indicated for posterior talofibular ligament injury. A summary of recommendations are presented in Table 25. It is important to emphasise that this information is a summary of the results and should be interpreted with consideration of the full results table (Table 24).

**Table 25:** Accuracy Summary - Musculoskeletal Clinical Indications for Diagnostic Ultrasound Imaging in the Ankle/Foot Region

Target Condition	Recommendation
Tendons and soft tissue	Grade
Anterior talofibular ligament injury	3
Calcaneofibular ligament injury	3
Peroneal tendon tears	3
Peroneal subluxation	3
Posterior tibial tendinopathy	3
Plantaris Tendon tears	3
Plantar fasciitis	3
Achilles tendinopathy	2
Deltoid ligament injury	2
Plantar plate tears	2
Syndesmotic Injury	2
Anterolateral ankle impingement	0
Posterior talofibular ligament injury	0
Bursitis	Unknown
Ganglion cyst	Unknown
Retinaculum pathology	Unknown
Tibialis anterior tendinopathy	Unknown
Nerves	
Morton’s neuroma	2
Tarsal tunnel syndrome	Unknown

Unknown: No diagnostic accuracy studies found

Grade 0: Not indicated

Grade 1: Conflicting evidence (test results should be interpreted with caution)

Grade 2: Equivalent to other imaging techniques (other techniques might provide significant information)

Grade 3: First choice technique (other techniques rarely provide more information)

## Discussion

Diagnostic ultrasound is a common imaging modality used to assist in the diagnosis of musculoskeletal complaints when the clinical picture is uncertain [34]. The aim of this study was to undertake a comprehensive review of the literature to assess the diagnostic accuracy of MSK-DUSI for the diagnosis of soft tissue pathology of the extremities. All musculoskeletal soft-tissue conditions identified by the ESMR and ACR MSK-DUSI guidelines were included in this review [3, 55]. This review does not cover the entire utility of this technology.

This discussion section is divided into sub-sections. These relate to the anatomical areas discussed in the results section and include shoulder, elbow, wrist/hand, hip, knee, and ankle/foot.

### Shoulder

Shoulder complaints are common in primary and secondary care settings [157, 158]. Most shoulder complaints present with similar signs and symptoms, often making a definitive diagnosis difficult. Even following a thorough history and physical examination there is often a significant degree of clinical uncertainty [159]. An accurate diagnosis is essential to ensure that patients receive appropriate and timely treatment and correct information regarding their prognosis. Overall, the results showed that MSK-DUSI was a useful imaging method to accurately detect certain musculoskeletal disorders of the shoulder. Importantly, the results demonstrated high discriminatory ability for detecting any rotator cuff tear and the ability to rule in rotator cuff atrophy with moderate to high accuracy. This is desirable, because surgical repair is sometimes required and positive post-operative outcomes have been correlated with early surgical repair [160, 161] and the absence of rotator cuff atrophy [162]. For partial thickness rotator cuff tears it is important to emphasise that it is easier to rule it in (SpC: 0.75 to 0.98; LR + = 1.84 to 35.5) than to rule it out (SnS: 0.46 to 0.84; LR- = 0.18 to 0.72).

Based on the results for the shoulder region it seemed that the use of MSK-DUSI is indicated for any rotator cuff tear, subacromial bursitis, calcific tendinitis, rotator cuff tendinopathy, rotator cuff atrophy, subacromial impingement syndrome and long head of the biceps pathology.

### Elbow

The utility of MSK-DUSI for the elbow has been well-described [3], however this review found limited diagnostic studies in this area, with the exception of lateral epicondylalgia and cubital tunnel syndrome. The results showed that hypoechogenity of the common extensor origin had the best combination of diagnostic SnS and SpC in determining elbows with lateral epicondylalgia. Other ultrasound features found in chronic cases include neovascularity, calcifications and cortical irregularities which show high SpC but very low SnS. There was little clarity on the role of these findings in the diagnosis of lateral epicondylalgia [76]. The use of MSK-DUSI is recommended as an objective tool to complement the clinical reference standard when the diagnosis is uncertain.

For cubital tunnel syndrome, the diagnostic value of MSK-DUSI showed a wide variation between the studies included in Beekman et al. [77] review. The wide range in diagnostic accuracy was likely due to a number of factors. Methodological flaws were present in most of the studies. In addition, there was no consensus on the ideal scanning procedure and no standardised cross-sectional area measurements to determine an abnormal ulnar nerve thickening at the elbow. It is likely that MSK-DUSI may be helpful in the diagnosis by demonstrating ulnar nerve thickening and by detecting underlying abnormalities. However, MSK-DUSI results should be interpreted with some caution due to its wide variation of diagnostic accuracy.

Based on the results for the elbow region it is recommended that the use of MSK-DUSI is indicated for objectively identifying lateral and medial epicondylalgia when the clinical picture is uncertain and full-thickness tears of the distal biceps tendon. It is likely that MSK-DUSI may be helpful in the diagnosis of cubital tunnel syndrome, however it is recommended to clinicians that they do not rely on negative test findings to rule it out and to use appropriate clinical judgement whether or not to follow up with electrodiagnostic studies.

### Wrist/hand

The results showed ultrasound diagnosis of carpal tunnel syndrome (CTS) was the most frequently investigated condition of the wrist/hand. CTS is typically diagnosed clinically, with electrodiagnostic studies (NCS and/or EMG) used to confirm its presence [81]. However, electrodiagnostic studies have limitations; they are uncomfortable and cannot directly assess the surrounding anatomy, which is why MSK-DUSI has emerged as a possible alternative diagnostic tool [82]. The results showed a wide variation of the diagnostic accuracy of MSK-DUSI in the assessment of median nerve cross-sectional area at the wrist. This variation might be explained by different scanning protocols and reference ranges for median nerve cross-sectional area, along with differences in study design (e.g. blinding, selection of patients and controls, retrospective or prospective study design). This review did not allow for strong conclusions to be made about the diagnostic accuracy of MSK-DUSI due to the wide variation of results, however the majority of studies demonstrated moderate SnS and SpC. It is the authors’ opinion that MSK-DUSI would appear to be complementary to electrodiagnostic studies rather than an alternative.

de Quervains disease is typically an easy clinical diagnosis with pain and tenderness in the first extensor compartment of the wrist and a positive Finkelstein test. [163] However, the presence of an intracompartmental septum, an anatomical variation, has been reported to increase the risk of non-operative treatment failure and thus prognosis [89]. Therefore, studies assessing the accuracy of MSK-DUSI for detecting this septum were included. The results showed MSK-DUSI had high diagnostic value for detecting the septum and findings associated with de Quervains disease. MSK-DUSI has high diagnostic value for ganglion cysts. However, the size of the lesion can influence the SnS of MSK-DUSI for detecting ganglion cysts. The classic ultrasound diagnostic criteria for ganglion cysts has been described as an anechoic (dark/black) mass with thin, relatively sharp borders and posterior acoustic enhancement (the area behind an anechoic structure appears more echogenic (brighter) than its surroundings) [164]. Recently, researchers have reported that small ganglion cysts (≤10 mm) appear hypoechoic without posterior acoustic enhancement and thus do not fulfil the normal criteria [164]. Future studies in this area should take into account both diagnostic criteria with an aim to minimise the potential of false negative findings.

The intrinsic wrist ligaments and triangular fibrocartilage complex (TFCC) can be assessed at least in part by MSK-DUSI. The two most important intrinsic wrist ligaments are the scapholunate (SLL) and lunotriquetral (LTL), as their disruption may result in significant pain, instability and loss of function [98]. MSK-DUSI can rule in SLL and LTL injury but cannot rule it out. Imaging these structures with MSK-DUSI requires dynamic manoeuvers which are difficult to reproduce which might explain ultrasounds lack of SnS [91]. Ulnar collateral ligament (UCL) ruptures are not uncommon but are easily misdiagnosed and mistreated in the primary care setting [90]. An accurate and safe method of diagnosis is typically required. The results showed MSK-DUSI had high diagnostic value for ulnar collateral ligament (UCL) injury (displaced and non-displaced). Due to the overall paucity of the literature for intrinsic wrist ligament and TFCC injury the results should be interpreted with some caution.

Based on the results for the wrist/hand region it is recommended that MSK-DUSI be used for de Quervains, ganglion cysts and any UCL tear. It is likely that MSK-DUSI might be helpful in the diagnosis of carpal tunnel syndrome and can be used as a screening tool. However, it is recommended to clinicians that they not rely on negative test findings to rule out carpal tunnel syndrome and to use appropriate clinical judgement whether or not to follow up with electrodiagnostic studies. With the current state of the technology the use of MSK-DUSI is not indicated for SLL, LTL or TFCC injury.

### Hip

The reported clinical indications for MSK-DUSI of soft tissue structures of the hip are great, varying from tendinopathy and tears to bursitis and snapping hip syndrome [3, 6]. This review found limited studies investigating the diagnostic value of MSK-DUSI for these and other soft tissue conditions of the hip. Hamstring injuries are among the most common in sports that involve sprinting and jumping, but are also common in dancing and water-skiing [165]. Both MSK-DUSI and MRI technologies have been advocated in cases of hamstring injury [166, 167], thus the author found the lack of diagnostic studies for this muscle region a notable gap in the literature. While both imaging modalities are considered useful in identifying hamstring injuries when oedema and haemorrhage are present [167], MRI is considered superior for evaluating injuries to deep portions of the muscles [168], or when a previous hamstring injury is present, as residual scarring can be misinterpreted on an ultrasound image as an acute injury [166]. Due to these factors MRI is considered to provide a more accurate diagnosis than MSK-DUSI [6].

Overall, the deep location of the target structures, complex anatomy, and extensive investigation area is challenging for the current capabilities of MSK-DUSI. Low to medium frequency transducers are required, providing increased image depth at the expense of resolution. In addition, the small field of view provided by the ultrasound exam limits the ability to exclude significant findings beyond the examined region [6]. These technical limitations of MSK-DUSI appear to favour MRI or CT for diagnostic purposes and may explain why a paucity of studies were found.

Based on the results for the hip region it is recommended that the use of MSK-DUSI is indicated for gluteal tendon tears, trochanteric bursitis and meralgia paresthetica.

### Knee

The results show MSK-DUSI has a high diagnostic value for full quadriceps tendon tears, Baker’s cysts and meniscal cysts. The medial patella plica (MPP) has been reported as the most commonly injured plica due to its anatomical location [169] and mimics the presentation of other internal derangements of the knee [170]. MSK-DUSI can rule out MPP syndrome with high accuracy and can rule it in with moderate accuracy. Stubbings et al. [170] found that the MPP test (orthopaedic test) and MSK-DUSI possesses superior diagnostic accuracy compared to MRI.

Patellar tendinopathy is typically an easy diagnosis based primarily on clinical examination, where it presents as activity-related anterior knee pain associated with well-localised, palpable patella tendon tenderness [120]. The results show MSK-DUSI has a moderate to high diagnostic value for patellar tendinopathy. However, a clinical question should be asked, ‘is further imaging necessary in the light that it will not change the treatment plan?’ MSK-DUSI may potentially have a role in assessing the severity of disease, thus prognosis and/or patient education, but this is yet to be established.

Posterolateral knee structures (LCL, popliteal tendon and popliteofibular ligament) along with meniscal and other ligament injuries of the knee should be grouped together when assessing the diagnostic value of MSK-DUSI. This is because injury to one of these structures rarely occurs in isolation but rather they occur in combination and often also with osseous involvement (fracture, bone bruise) [171–175]. The results show a wide variation in the capability of MSK-DUSI to detect these structures accurately. This might be explained by a technical factor: the required increased depth of penetration is obtained at the expense of image resolution. It also seems reasonable to assume that the accuracy of MSK-DUSI may be influenced by an expanding haemarthrosis, which is commonly associated with internal knee derangement [116]. This again requires increased depth of penetration and results in decreased image resolution (i.e. ultrasound accuracy may decrease with increasing time between knee injury and the ultrasound examination). A major limitation of MSK-DUSI of the knee menisci is the inability to visualise the entire meniscus, due to the presence of artifacts and difficulty in imaging the inner margins if the meniscus [103]. The diagnostic value of MSK-DUSI for ligamentous and meniscal lesions relies on its ability to visualise all of these structures as they often occur in combination. The results highlight that the diagnostic accuracy of MSK-DUSI varies between each condition thus limiting its usefulness as a primary imaging modality for suspected internal knee derangement. However, this does not mean MSK-DUSI does not have a role to play in assessing internal knee derangement. Its potential use is as a side-line, point-of-care screening tool at sporting events rather than a definitive diagnostic tool in a primary or secondary care setting, but this is yet to be established.

The results show MSK-DUSI has high diagnostic accuracy in ruling common peroneal neuropathy out but is less sensitive in ruling it in. Electrodiagnostic studies have been reported to have a false negative rate of up to 30 % for this condition [118]. However, it is importanto emphasise that MSK-DUSI was not introduced to replace electrodiagnostic investigation of common peroneal neuropathy but to act as a complementary modality to assess nerve cross-sectional area to improve diagnostic accuracy and to assess for potential structural causes [118].

Based on the results for the knee region it is recommended that the use of MSK-DUSI is indicated for full-thickness quadriceps tendon tears, patella tendinopathy, medial patella plica syndrome, Baker’s cysts and meniscal cysts. It is likely that MSK-DUSI may be helpful in the diagnosis of common peroneal nerve neuropathy and can be used as a screening tool. However, it is recommended to clinicians that they do not rely on negative test findings to rule out common peroneal nerve neuropathy and to use appropriate clinical judgement whether or not to follow up with electrodiagnostic studies. With the current state of the technology the author recommends that the use of MSK-DUSI is not indicated as a definitive diagnostic tool for ligamentous and meniscal tears of the knee, however may have a role as a side-line, point-of-care screening tool at sporting events. MSK-DUSI is not indicated for partial-thickness ACL tears.

### Ankle/foot

Ligament and syndesmotic injuries are common and some patients develop functional instability, persistent pain and swelling [124, 132]. With prompt, accurate grading of the injury the appropriate conservative or surgical management can be taken. Early, appropriate management has been shown to reduce the risk of developing chronic ankle instability symptoms by 70-90 % [176]. Of the ligamentous structures of the ankle/foot MSK-DUSI has high diagnostic value for anterior talofibular ligament, calcaneofibular ligament, deltoid ligament, posterior talofibular ligament and syndesmotic injury. The high accuracy of posterior talofibular ligament injury was based off one subject, therefore the use of MSK-DUSI for this condition is not recommended due to lack of current evidence.

The results show MSK-DUSI has high diagnostic value for plantaris tendon tears, peroneal tendon tears and posterior tibial tendinopathy. Tendinosis, tendinitis, peritendinitis, and partial or complete tendon rupture are all causes of achilles tendinopathy [137]. The value of MSK-DUSI lies within its ability to differentially diagnose these causes as shown by the results. It has been reported that the chronicity of Achilles tendon tears might impact the SnS of MSK-DUSI because fibrous scarring and granulomatous tissue can mask the defect and is therefore often overlooked [134]. Overall, the results show MSK-DUSI has moderate to high diagnostic value for differentiating Achilles tendinopathy.

The diagnosis of Morton’s neuroma is typically clear with a thorough history and physical examination. Clinical suspicion should arise if the patient gives a history of pain or tingling on the plantar aspect of the foot, made worse whilst wearing tight shoes and relieved by rest. Clinical examination may reveal tenderness on direct palpation, squeezing the metatarsals together or on stretching toes around the affected web space, a feeling of reduced sensation in between the toes of the affected area or a ‘Mulder’s Click’ [126, 148]. However, in cases of doubtful symptomatology and double lesions imaging studies may be indicated [154]. The results show MSK-DUSI has high diagnostic value for Morton’s neuroma. Anterolateral impingement syndrome can occur from a variety of causes including ankle instability, osseous and soft tissue changes [142]. Therefore, MSK-DUSI has emerged has a non-invasive tool to detect the presence of a soft tissue lesion as the cause [125]. There is conflicting evidence for the use of MSK-DUSI, therefore the results should be interpreted carefully. The difference in diagnostic accuracy is likely due to the heterogenic study population and study size. Cochet et al. [125] report on 41 subjects from the general population whereas McCarthy et al. [142] reported on 17 subjects from a population of elite athletes.

Based on the results for the ankle/foot region it is recommended that the use of MSK-DUSI is indicated for anterior talofibular ligament injury and for Morton’s neuroma when the clinical picture is uncertain. To a lesser extent MSK-DUSI can diagnose calcaneofibular and deltoid ligament injury; syndesmotic injury, plantaris tendon tears, peroneal tendon tears, posterior tibial tendinopathy, peroneal subluxation and plantar fasciitis. MSK-DUSI is recommended for differentiating causes of Achilles tendinopathy. However, a negative test may need to be followed up with MRI if the patient fits the clinical picture for a partial tear. With the current state of the technology the author recommends that the use of MSK-DUSI is not indicated for plantar plate tears, posterior talofibular ligament tears and anterolateral ankle impingement.

#### Comparison with existing reviews

To the authors knowledge this is the first time a review has examined the accuracy of MSK-DUSI to diagnose a full spectrum of musculoskeletal soft-tissue disorders of the upper and lower limb. Only one other study relating to the spectrum of conditions in this review was identified. The paper by Klauser et al. [3] was a combined review and Delphi consensus. Klauser et al. [3] did not report quantitative diagnostic accuracy data (SnS; SpC; LRs). A limitation of Klauser et al. [3] study. Rather, Klauser et al. [3] reported the evidence level (Level A: consistent randomised controlled clinical trial or prospective cohort study; Level B: Consistent retrospective cohort, exploratory cohort or case–control study; Level C: case series study) combined with the final Delphi consensus (grade/strength of recommendation from 0 to 3, with grade 3: ultrasound is the first choice level technique).

In this article, the review was limited to the inclusion to musculoskeletal soft tissue conditions identified by the ESMR and ACR MSK-DUSI guidelines [3, 55]. Whereas Klauser et al. [3] included all clinical indications for MSK-DUSI (i.e. soft tissue, nerve, osseous and joint pathology). A considerable strength of this article was the reported quantitative diagnostic accuracy data for each individual study and when appropriate the provided pooled data. A key comparison of this review and Klauser et al. [3] paper should be emphasised. The clinical conditions where MSK-DUSI was found to have moderate to high diagnostic accuracy in this review consistently matched Klauser et al. [3] final Delphi consensus with a grade/strength of recommendation of 2 or 3.

#### Strengths and weaknesses of the review

The results were based on a comprehensive and sensitive literature search strategy that aimed to identify all relevant systematic reviews of diagnostic studies, all diagnostic studies published after the date of the latest systematic reviews and relevant diagnostic studies outside the scope the systematic reviews in the National Library of Medicine’s PubMed data base (1972 to mid-2014). Wide search terms, not limited by language were used, and retrieved reference lists were manually searched for relevant primary studies to include in the review. In addition, to the authors knowledge this is the first time a review has examined the accuracy of MSK-DUSI to diagnose a full spectrum of musculoskeletal soft-tissue disorders of the upper and lower extremity. It is the authors’ opinion that the scope and breadth of the review is a strength in itself and most importantly a strength to the reader. In particular, the comprehensive range of accuracy statistics is a significant strength.

It is important to emphasise that this study is not a systematic review and is instead a narrative review. Although we used a comprehensive literature search strategy our search may not have been completely exhaustive, however if relevant studies were missed they were likely few in number and would be unlikely to impact the results with any significance. Clinical indications for which this report concludes the evidence currently shows MSK-DUSI has moderate to high diagnostic accuracy or even low diagnostic accuracy sometimes rests on a single diagnostic study. The quality of the diagnostic study also has a substantial influence on the conclusions.

Other potential weaknesses of this review include that there was only one reviewer in the selection of the studies for inclusion. When two or more independent, blinded reviewers select studies for inclusion and then independently extract data the potential for bias decreases. This review included all types of diagnostic studies, including retrospective studies. It has been shown that retrospective data is associated with an overestimation of results [177]. The reviewer did a fundamental appraisal of the methodological quality of studies, as outlined by the Users’ Guide to the Medical Literature: A Manual for Evidence-Based Clinical Practice but did not use a study quality assessment tool such as the Standards for Reporting Studies of Diagnostic Accuracy (STARD) or the Quality Assessment of Diagnostic Accuracy Studies (QUADAS) criterion lists [178]. While critical appraisal of the included reviews and diagnostic studies would be ideal, it was beyond the scope of the present report. It was noted during this process that the strength of evidence for a large proportion of test comparisons were limited because most studies were small, heterogeneous and had design flaws, thus potentially limiting the reliability of their findings.

In about half the studies not all patients who had an ultrasound scan (index test) underwent the reference test allowing for potential verification bias to affect the results. This often occurs as the reference standard is usually invasive, expensive or both and the issue then becomes ethical in nature. The mean time from injury to ultrasound was poorly reported in about two in three studies, therefore it was not possible to compare between acute and chronic complaints. Consequently, disease progression bias might have influenced the results. However, it has been reported that these design flaws have minimal effect on estimates of diagnostic accuracy [177]. The articles assessing rotator cuff tears did not differentiate between specific structures, as such we could not evaluate each rotator cuff separately. The MSK-DUSI criteria used to consider that there was a full or partial-thickness rotator cuff tear was not reported in all studies and may have differing definitions between studies. It is well-documented that the effective use of diagnostic ultrasound is highly dependent on operator skill and training [14, 179]. Unfortunately, most studies did not state the experience level of non-radiologists. These design flaws of the original studies may have influenced the reliability of their findings.

It is important to emphasise that the vast majority of studies were conducted in a secondary care setting. In addition, about 70 % of the studies had a surgical reference standard. This implies that these studies may have included a high proportion of more severe cases and therefore, it is uncertain whether the diagnostic value of MSK-DUSI will be similar when used in primary care settings. Due to the limitations discussed, clinicians should interpret the results with some caution because of the potential for overestimation of diagnostic accuracy.

#### Future research

There is a lack of high quality prospective experimental studies that directly compare the accuracy of MSK-DUSI for soft-tissue pathology of the extremities to an appropriate reference standard. Consequently, future research should focus on prospective experimental studies to reduce the potential risk of spectrum and verification bias. The vast majority of studies were conducted in a secondary care setting, thus limiting the ability to generalise the results to a primary care setting. With the growth of MSK-DUSI among non-radiologists in a primary care setting [31] future studies are needed in order to evaluate the accuracy of MSK-DUSI in a primary care setting and with operators and reviewers who are not musculoskeletal radiologists.

It seems apparent that the lack of standardised values of abnormal nerve cross-sectional area impact significantly on MSK-DUSI diagnostic accuracy studies. Consequently, future research should be undertaken to standardise normal and abnormal nerve cross-sectional area values before further research is taken in investigating the diagnostic value of MSK-DUSI. Other areas of research for individual conditions were also noted. For example, MSK-DUSI has high diagnostic value for patella tendinopathy. This condition is a simple clinical diagnosis and is always treated conservatively, thus the value in imaging needs to be questioned if it does not change management. This example applies to several conditions in this review. Research in this area should focus on investigating whether MSK-DUSI can demonstrate the severity of disease, thus potentially determine prognosis and track the response to treatment. Furthermore, research in this area could involve MSK-DUSI imaging for patient education and its effect on clinical outcomes. In addition, this review found over 30 clinically indicated conditions with no diagnostic accuracy studies. Overall, there is a lack of high quality literature on the diagnostic accuracy of MSK-DUSI for a wide variety of clinically indicated conditions and future research should be considered a high priority.

## Conclusion

The purpose of this article was to undertake a structured review of the literature to assess the accuracy of diagnostic ultrasound for the diagnosis of musculoskeletal soft tissue pathology of the extremities. The results of this review indicated that MSK-DUSI has good diagnostic accuracy for the detection of a wide spectrum of soft tissue conditions of the extremities. As such, MSK-DUSI is recommended as a non-invasive, relatively cheap, accurate, quick and accessible imaging modality for a variety of soft tissue conditions of the extremities. However, the current evidence base presents with some limitations. Overall, there is a lack of high quality literature on the diagnostic accuracy of MSK-DUSI for a variety of clinically indicated conditions and future research should be considered a high priority.

## Additional file

Additional file 1:
**Search Terms - full electronic search strategy.** (PDF 84 kb)

## Abbreviations

2D: 2-dimensional
3D: 3-dimensional
CT: Computed tomography
EMG: Electromyography
LCL: Lateral collateral ligament
LR-: Negative likelihood ratio
LR+: Positive likelihood ratio
LTL: Lunotriquetral ligament
MPP: Medial patella plica
MRA: Magnetic Resonance Arthrography
MRI: Magnetic Resonance Imaging
MSK-DUSI: Musculoskeletal diagnostic ultrasound imaging
MSK-US: Musculoskeletal ultrasound imaging
NCS: Nerve conduction study
PET: Positron emission tomography
QUADAS: Quality Assessment of Diagnostic Accuracy Studies
RUSI: Rehabilitative ultrasound imaging
SLL: Scapholunate ligament
SnS: Sensitivity
SpC: Specificity
STARD: Standards for Reporting Studies of Diagnostic Accuracy
TFCC: Triangular fibrocartilage complex
UCL: Ulna collateral ligament
