# Supplementary material for: The accuracy of diagnostic ultrasound imaging for musculoskeletal soft tissue pathology of the extremities: a comprehensive review of the literature
Source: Chiropr Man Therap. 2015 Nov 5;23:31. doi: 10.1186/s12998-015-0076-5 (PMC4634582; doi:10.1186/s12998-015-0076-5)
Supplement: Additional file 1: — Search Terms - full electronic search strategy. (PDF 84 kb) [file 12998_2015_76_MOESM1_ESM.pdf]

Additional file 1 Search Terms

**Search Terms<br>Index test**	**Search Terms<br>Diagnostic Accuracy**	**Search Terms<br>Target Condition/Region**
Ultrasonography (MeSH Term)	Sensitivity and Specificity (MeSH Term)<br>Accuracy	Shoulder (MeSH Term)<br>Elbow Joint (MeSH Term)<br>Wrist Joint (MeSH Term)<br>Hand (MeSH Term)<br>Hip (MeSH Term)<br>Knee Joint (MeSH Term)<br>Ankle Joint (MeSH Term)<br>Foot (MeSH Term)<br>Tendons (MeSH Term)<br>Ligaments (MeSH Term)<br>Muscles (MeSH Term)<br>Soft Tissue Injuries (MeSH Term)<br>Nerve Compression Syndromes (MeSH Term)<br>Tendinopathy (MeSH Term)<br>Tears (MeSH Term)<br>Bursitis (MeSH Term)

Example:

1) ((ultrasonography) AND (sensitivity and specificity)) AND shoulder

2) ((ultrasonography) AND accuracy) AND shoulder

The example shown above was repeated with each target condition/region.
